# Identification of potential blood biomarkers associated with suicide in major depressive disorder

**DOI:** 10.1038/s41398-022-01918-w

**Published:** 2022-04-14

**Authors:** Firoza Mamdani, Matthieu D. Weber, Blynn Bunney, Kathleen Burke, Preston Cartagena, David Walsh, Francis S. Lee, Jack Barchas, Alan F. Schatzberg, Richard M. Myers, Stanley J. Watson, Huda Akil, Marquis P. Vawter, William E. Bunney, Adolfo Sequeira

**Affiliations:** 1 Psychiatry and Human Behavior, University of California, Irvine, CA USA; 2 Department of Psychiatry, Weill Cornell Medical College, New York, NY USA; 3 Department of Psychiatry and Behavioral Sciences, Stanford University, Palo Alto, CA USA; 4 Hudson Alpha Institute for Biotechnology, Huntsville, AL USA; 5 Molecular & Behavioral Neuroscience Institute, University of Michigan, Ann Arbor, MI USA

**Keywords:** Personalized medicine, Molecular neuroscience

## Abstract

Suicides have increased to over 48,000 deaths yearly in the United States. Major depressive disorder (MDD) is the most common diagnosis among suicides, and identifying those at the highest risk for suicide is a pressing challenge. The objective of this study is to identify changes in gene expression associated with suicide in brain and blood for the development of biomarkers for suicide. Blood and brain were available for 45 subjects (53 blood samples and 69 dorsolateral prefrontal cortex (DLPFC) samples in total). Samples were collected from MDD patients who died by suicide (MDD-S), MDDs who died by other means (MDD-NS) and non-psychiatric controls. We analyzed gene expression using RNA and the NanoString platform. In blood, we identified 14 genes which significantly differentiated MDD-S versus MDD-NS. The top six genes differentially expressed in blood were: PER3, MTPAP, SLC25A26, CD19, SOX9, and GAR1. Additionally, four genes showed significant changes in brain and blood between MDD-S and MDD-NS; SOX9 was decreased and PER3 was increased in MDD-S in both tissues, while CD19 and TERF1 were increased in blood but decreased in DLPFC. To our knowledge, this is the first study to analyze matched blood and brain samples in a well-defined population of MDDs demonstrating significant differences in gene expression associated with completed suicide. Our results strongly suggest that blood gene expression is highly informative to understand molecular changes in suicide. Developing a suicide biomarker signature in blood could help health care professionals to identify subjects at high risk for suicide.

## Introduction

Suicide is a serious global public health problem that accounts for close to 800,000 deaths per year [1]. In the United States alone, suicide rates increased by more than 35% over the past 20 years, and in the past year, over 48,000 Americans died by suicide, making it the 10th leading cause of death [2]. Suicide prevention strategies and current medications, although helpful, have not stemmed the increase in self-inflicted deaths. Many individuals do not disclose suicidal intentions despite frequent contact with health care professionals. An estimated 30% of suicides visit a healthcare provider within a month of the suicide event [3, 4]. A dramatic rise in suicide also occurs in the days to weeks following discharge from psychiatric hospitals [5–7]. Thus, there is a critical opportunity for healthcare providers to evaluate at-risk individuals with a blood biomarker test to assess serious suicide intent.

Major depressive disorder (MDD) is the most common psychiatric diagnosis in suicide although the underlying biological determinants have yet to be identified. Converging evidence suggests that suicide is associated with a dysregulated stress response system and concurrently, an increase in inflammatory responses and their relevant downstream effects [8]. Additional clues to suicidality come from postmortem investigations in brain from depressed suicides showing disruptions in glucocorticoid and inflammatory responses [9–13], excitatory and inhibitory neurotransmitters (glutamate and GABA) [11, 14, 15], as well as dysregulation of polyamine stress response [16–24]. Expression of polyamine related genes in particular has consistently been shown to be altered in suicides compared to controls. The first study to specifically evaluate gene expression in DLPFC from depressed suicides (MDD-S, *N* = 21), depressed non-suicides (MDD-NS, *N* = 9) and controls (*N* = 29) using RNA-Seq found significant alterations of the spermidine/spermine N1-Acetyltransferase1 (SAT1) gene in both MDD groups [16]. A similar study reported dysregulation of SAT1 and significant alterations in glial cells in MDD-S [25]. In translational studies, SAT1 expression in blood was shown to be a potential peripheral suicide biomarker in males [26, 27], but not in females [28] suggesting a sex-specific effect that is not often taken into account when studying suicide.

Although many investigations focused on postmortem brain, to our knowledge there are no studies comparing gene expression in both postmortem blood and brain in samples taken from the same individuals. In this study we analyzed gene expression in MDD patients who died by suicide (MDD-S), in MDD patients who died of other causes (MDD-NS) and in non-psychiatric controls (C). The objective of our study is to provide preliminary information for the development of a blood biomarker signature that can be used to identify MDD patients at high risk for suicide.

## Materials and methods

### Subjects and samples

Brain and blood samples were obtained from the University of California, Irvine (UCI)-Pritzker Brain Bank per the Institutional Review Board (IRB). Consent was obtained from next-of-kin. Samples consisted of archival blood and dorsolateral prefrontal cortex (DLPFC) from MDD-suicides (*N* = 24 brain, 19 blood), MDD-non-suicides (*N* = 24 brain, 18 blood), and non-psychiatric controls (*N* = 21 brain, 16 blood). A psychological autopsy, through an extensive review of multiple sources of information, was performed by trained clinicians as described elsewhere [15, 29, 30]. Table 1 and Supplementary Table 1 list demographics of the subjects, including gender, age, diagnosis, race, blood/brain availability, cause of death, and clinical characteristics.

**Table 1:** Sample demographics for the blood and brain cohorts included in the study.

Sample	Diagnosis	*N*	Gender		Age		Education (Years)	Sleep disturbances (*N*)	Hx psych hosp (*N*)	Hx suicide attempt (*N*)	Suicidal ideation (*N*)
			Males	Females	Males	Females					
Brain	Control	21	17	4	45.71	47.50	13.40	2	0	0	0
	MDD-NS	24	11	13	50.73	50.92	13.83	13	8	5	10
	MDD-S	24	11	13	42.82	37.83	12.82	14	10	11	22
Blood	Control	16	12	4	43.08	45.20	13.73	2	0	0	0
	MDD-NS	18	11	7	51.45	55.57	14.06	11	8	5	7
	MDD-S	19	12	7	42.83	36.57	13.05	11	10	10	17

*MDD-S* major depressive disorder—suicide, *MDD-NS* major depressive disorder—non-suicide, *N* number of samples, *Hx* history, *Psych Hosp* psychiatric hospitalization.

Postmortem blood samples were collected in EDTA tubes from the heart or femoral vein and stored in −20 °C freezers until RNA extraction. In some cases, only brain tissue was available (coagulation sometimes prevents blood draw) while only blood was available for some subjects due to consent preferences by the next-of-kin (Supplementary Table 1). The human brain dissection and freezing protocol is described in detail in Supplementary Information. Briefly, the whole brain is first inspected and photographed before the brainstem and cerebellum are separated from the cerebrum. The cerebrum is coronally sliced from anterior to posterior and slices are placed on steel plates, labeled, photographed, and flash-frozen using cooled 2-methyl butane (−40 °C). Once frozen the brain slices are bagged and vacuum-sealed, then stored in −80 °C freezers until time for further dissections or extractions.

### RNA extractions

RNA extraction from blood was performed using the QIAamp RNA blood mini kit (Qiagen, Carlsbad, CA) and RNA extractions for brain samples were carried out using the Qiagen AllPrep DNA/RNA/Protein mini kit (Qiagen, Carlsbad, CA), both according to the manufacturer’s protocol. RNA concentrations were determined using Qubit fluorometric quantification (Thermo Fisher Scientific, Carlsbad, CA). Integrity of total RNA was evaluated using 18S and 28S ratios and the RNA integrity number (RIN) from the 2100 Agilent Bioanalyzer (Agilent technologies, Santa Clara, CA). RINs for both brain and blood are illustrated in Supplementary Fig. 1. RNA quality measures [i.e., pH, RIN, and postmortem interval (PMI)] for the brain samples are outlined in Supplementary Table 2 showing averages and standard deviations per group. Additionally, FOV (fields of view) were compared between brain and blood samples. The FOV is an imaging quality control measure from NanoString, which is indicative of the number of mRNA counts successfully imaged per lane in the array. Samples having low assay efficiency due to low quality of RNA will have a low number of counts and will result in a quality flag. The FOV threshold is set at 75% and any sample below this threshold is flagged and should not be used for downstream analysis.

### NanoString assays for blood and brain

RNA (5 µl at a concentration of 20 ng/µl) was directly quantified using the NanoString nCounter analysis system (NanoString, Seattle, WA). The NanoString platform allows, without reverse transcription, to directly and digitally quantify the number of mRNA molecules present in a sample in a highly multiplexed manner within a single reaction using colored molecular barcodes. NanoString is less sensitive to RNA degradation and factors affecting RNA quality, such as sample degradation, fixing procedures, postmortem interval, and pH because RNA is directly assayed without reverse transcription or amplification [31–33]. This platform was therefore particularly useful to assay gene expression in postmortem archival blood samples. Samples were processed at the Genomics High-Throughput Facility of the University of California, Irvine. NanoString probes were custom designed to include house-keeping genes and 114 genes of interest for depression and suicide, including genes within the polyamine and metallothionein systems, stress response, glutamatergic and GABAergic neurotransmission, immune response, circadian rhythm, mitochondria function, and telomere length. Gene targets, accession number, probe position, and supporting references are outlined in Supplementary Table 3.

### Statistical analysis

NanoString mRNA counts were processed using nSolver analysis software version 4.0 from NanoString for quality control (probe binding, counts, and image quality). The housekeeping genes included in our custom assay, detailed in Supplementary Table 3, included GAPDH, CYC1, and SDHA. Normalization procedures were done using the nCounter Analysis system global method, suggested by NanoString, that uses all probes or features to calculate a normalization factor instead of using a small number of housekeeping genes. Counts were logged (base 2) and imported into Partek Genomics Suite (Partek Inc, St-Louis, USA) for statistical analysis. ANOVA was used to investigate the effect of demographic variables, pH, and PMI on log_2_ gene expression levels. Gene expression and clinical phenotypic data were analyzed using multiple linear regression correcting for gender, postmortem factors, and age. The effects of suicide status, diagnosis and demographic variables (age, gender, and PMI) on gene expression were investigated using a 2-way ANCOVA model. For the DLPFC and blood, the influence of the time of death (TOD) on suicide and gene expression was assessed by adjusting the TOD to hours from sunrise as a continuous variable in the model to measure possible circadian effects. The adjusted TOD had a significant effect in the DLPFC, particularly for the expression of clock genes and was therefore included in the final analyses reported in the results section. Pair-wise post-hoc analyses were further performed to determine genes associated with suicide in MDD (MDD-S vs. MDD-NS). Other comparisons included the control group and both MDD groups (Control vs. MDD-NS and Control vs. MDD-S) as well as a comparison between the control and both MDD groups regardless of the suicide status Control vs. MDDs (non-suicides and suicides). To correct for multiple testing, a Benjamini–Hochberg false discovery rate (FDR) correction was used to generate adjusted *q*-values [34]. An estimate of the rate of the probability of a significant gene being a false discovery was calculated taking into account the number of comparisons (3 comparisons × 78 genes that were present both in blood and brain = 234 comparisons). A threshold of *q* < 0.1 was used for significance based on previous similar gene expression studies [16, 25] but focus on *p*-values was favored as this is an exploratory analysis of potential biomarkers to be confirmed in future studies.

We also sought to cross-validate our differentially expressed gene results with previous studies aiming to find suicide biomarkers in patients with various psychiatric disorders (MDDs, bipolar disorder and schizophrenia) using post-mortem and clinical blood [35]. We pooled our list of differently expressed genes between MDD-S and MDD-NS in blood/brain and compared our results to Niculescu and colleagues [35] Bonferroni-corrected lists of suicide biomarkers, which they validated in post-mortem samples. These analyses have a caveat in that our results are specific to MDD patients and we used a targeted multiplexed gene expression assay, while Niculescu et al. [35] used high throughput microarrays to study a number of psychiatric diagnoses (attention deficit disorder, anxiety disorder, bipolar disorder, MDD, mood disorder, not otherwise specified, psychosis not otherwise specified, post-traumatic stress disorder, schizoaffective disorder, and schizophrenia).

## Results

No significant differences were observed for pH or RIN between MDD-S, MDD-NS, and Controls for blood and DLPFC. Gender and age varied between groups and were included as covariates. The DLPFC (but not blood) showed an effect of time of death (“hours since sunrise”) and was included as a covariate in the DLPFC analyses (Supplementary Table 4). The quality of the RNA from post-mortem blood samples was lower than the brain RNA quality as expected. RINs for the blood RNA were low for a majority of the subjects and high in brain for most of the subjects (Supplementary Fig. 1) unsurprisingly as we used RNA extracted from non-preserved blood. The NanoString platform and additional data quality measures were used to make sure blood RNA could be used to obtain reliable gene expression data. Specifically, the fields of view (FOV), an imaging quality control measure indicative of the number of mRNA counts imaged per lane of the array were compared between brain and blood samples (Supplemental Table 5). The percent FOV was comparable between brain and blood and exceeded the 75% threshold set by NanoString, thus demonstrating the lack of effect of low RIN in the blood on mRNA measures.

### Suicide blood biomarkers

**Fig. 1:**
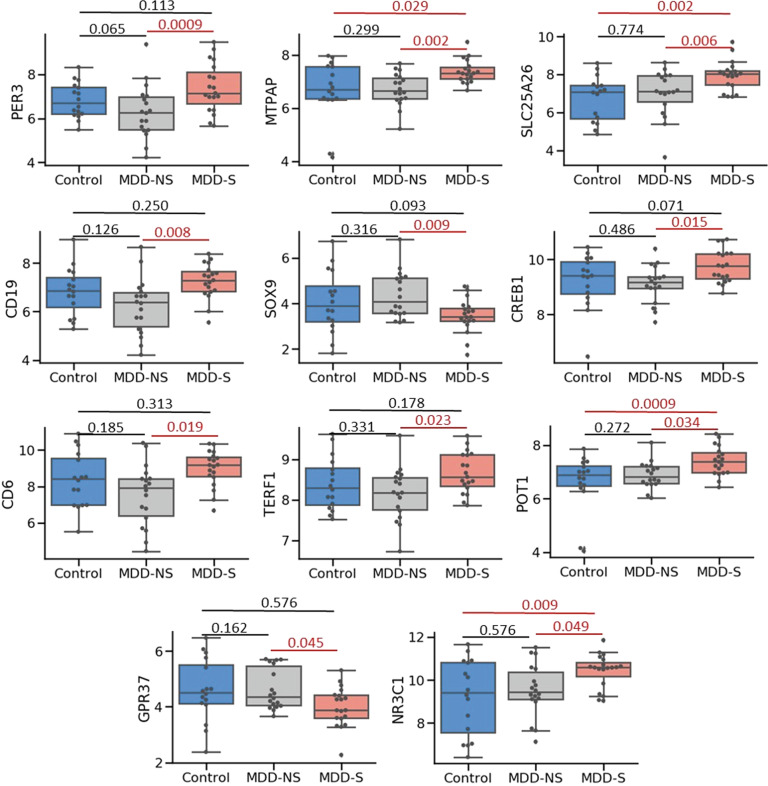
Genes differentially expressed in blood. Graphical representation of eleven genes significantly differentially expressed between MDD-S and MDD-NS in blood, at *p*-value ≤ 0.05, and also passing FDR correction for multiple testing at *q* ≤ 0.1. Four of the 11 genes; MTPAP (Mitochondrial Poly(A) Polymerase), SLC25A26 (Solute Carrier Family 25 Member 26), NR3C1 (Nuclear Receptor Subfamily 3 Group C Member 1), and POT1 (Protection of Telomeres 1) were also significantly differentially expressed between MDD-S and control groups.

Our main objective was to identify possible blood biomarkers for suicide comparing MDD-S and MDD-NS. As shown in Table 2, 14 genes significantly differentiated MDD-S from MDD-NS, at *p* ≤ 0.05, and eleven of these genes survived FDR correction at *q* ≤ 0.1, which is depicted in Fig. 1. Of these genes, two circadian genes, period circadian regulator 3 (PER3) and PER2 were significantly upregulated in MDD-S vs. MDD-NS (PER3: *p* = 0.001, *Q* = 0.1; FC = 2.650; PER2: (*p* = 0.041, *q* = 0.207; FC = 1.871). Additional differentially expressed genes (DEGs) included MTPAP (Mitochondrial Poly(A) Polymerase), SLC25A26 (Solute Carrier Family 25 Member 26), CD19 (B-Lymphocyte Antigen CD19), and GAR1 (GAR1 Ribonucleoprotein) while SOX9 (SRY-Box Transcription Factor 9) was decreased in suicides vs non-suicide MDDs.

**Table 2:** Blood-based significantly differentially expressed genes (*p* ≤ 0.05) between MDD-S and MDD-NS, the corresponding *q* value after FDR correction (*Q* ≤ 0.1 are in bold), and fold change in MDD-S relative to MDD-NS.

Gene	Mean (Control)	Mean (MDD-NS)	Mean (MDD-S)	*p* (MDD-S vs. MDD-NS)	*q* value (MDD-S vs. MDD-NS)	FC (MDD-S vs. MDD-NS)	FC direction
PER3	6.862	6.226	7.395	0.0009	**0.0617**	2.650	UP
MTPAP	6.824	6.628	7.377	0.002	**0.030**	1.801	UP
SLC25A26	6.834	6.815	7.945	0.006	**0.062**	2.119	UP
CD19	6.927	6.101	7.246	0.008	**0.050**	1.901	UP
SOX9	4.079	4.493	3.457	0.009	**0.050**	−2.0994	DOWN
GAR1	6.209	6.227	6.734	0.009	0.130	1.436	UP
CREB1	9.292	9.118	9.755	0.015	**0.050**	1.609	UP
CD6	8.542	7.553	8.984	0.020	**0.050**	2.346	UP
TERF1	8.445	8.106	8.667	0.023	**0.067**	1.411	UP
POT1	6.624	6.886	7.403	0.034	**0.051**	1.484	UP
PER2	5.156	4.774	5.541	0.041	0.207	1.871	UP
GPR37	4.513	4.600	3.996	0.043	**0.067**	−1.571	DOWN
MTHFR	8.928	8.673	9.034	0.045	0.130	1.408	UP
NR3C1	9.315	9.465	10.435	0.049	**0.067**	1.898	UP

*MDD-S* major depressive disorder—suicide, *MDD-NS* major depressive disorder—non-suicide, *FC* fold-change, *FDR* false discovery rate.

Two lymphocyte markers, CD19 and CD6, were upregulated MDD-S vs. MDD-NS (Fig. 1 and Table 2). CD19 is a surface membrane antigen of B-Lymphocytes used as a biomarker for lymphocyte development [36, 37]. Increased CD19+ B cells have been previously reported to be associated with depression [38]. CD6 is a surface membrane antigen of T-lymphocytes involved in the activation of T cells [39], and considered a promising target for autoimmunity and neuroinflammation [40]. CD6 is implicated in T cell infiltration into the central nervous system due its role in T cell trafficking across endothelial cell barriers [41]. Genetic variants of CD6 are associated with clinical response to TNF-alpha (Tumor Necrosis Factor-alpha) [42].

Twenty genes in blood (*p*-value ≤ 0.05; *Q*-value ≤ 0.1) were differentially expressed between MDD-S and controls (Supplementary Table 6). Interestingly, many of these genes overlapped with those identified between MDD-S and MDD-NS, including SLC25A26, GAR1, MTPAP, and NR3C1 (glucocorticoid receptor; Nuclear Receptor Subfamily 3 Group C Member 1). Also, of note is the significant difference in SAT1 gene expression in suicides. This gene has been associated with suicide and depression in postmortem brains and clinical populations with suicidal ideation [16, 24, 26, 43]. Comparisons were also carried out between MDD-NS and Controls (Supplementary Table 7), and an overall comparison between MDDs (MDD-S and MDD-NS) and Controls (Supplementary Table 8).

### Postmortem DLPFC gene expression

**Fig. 2:**
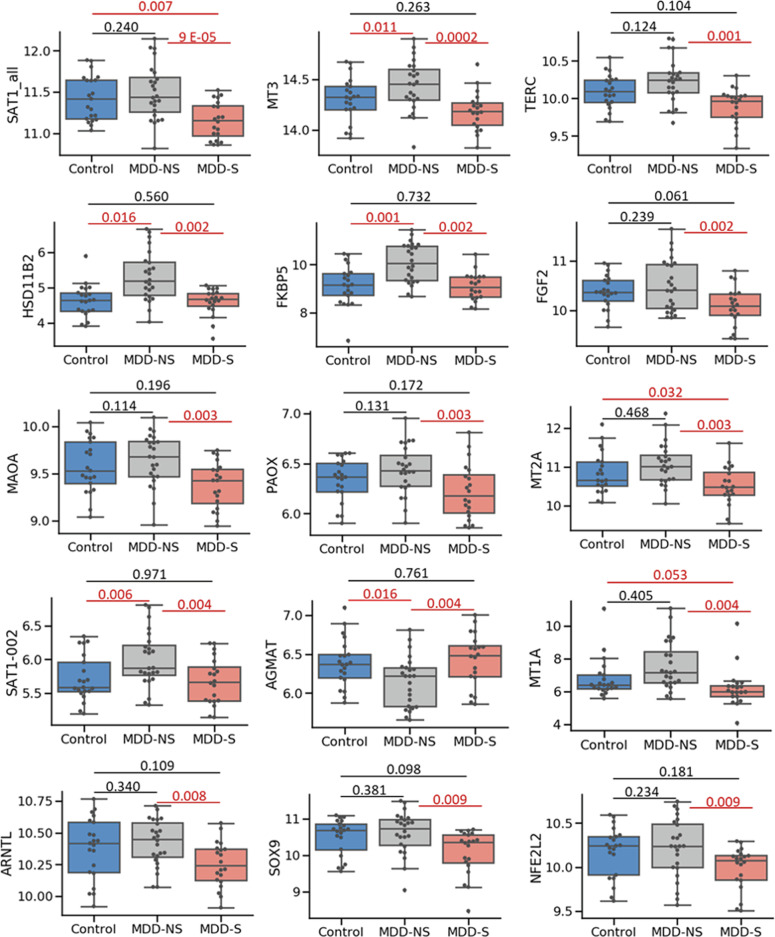
Genes differentially expressed in brain. Top fifteen (*p*-value ≤ 0.01) differentially expressed genes between MDD-S and MDD-NS in DLPFC. The displayed genes all passed FDR correction for multiple comparisons at *Q* ≤ 0.1.

A secondary objective of the current study was to investigate gene expression differences relevant to suicide in postmortem brain. Of the genes analyzed in the DLPFC, seventeen genes were downregulated, while only one gene (AGMAT) was upregulated in MDD-S compared to MDD-NS (at *p*-value ≤ 0.01, *Q*-value ≤ 0.1 (for 16 of the genes)) (Fig. 2 and Table 3). The top gene differentially expressed between MDD-S and MDD-NS was SAT1. In addition to the main SAT1 probe (SAT1_all), measuring all splice variants of the SAT1 gene, two additional splice variant specific probes (SAT1-002 and SAT1-001) were also significantly reduced in MDD-S (Fig. 2 and Table 3). The second most significant gene between MDD-S and MDD-NS was the brain-specific, MT3 (Metallothionein 3) gene, which was decreased in suicides. A total of four metallothioneins (MT3, MT2A, MT1A, and MT1X) were significantly reduced in MDD-S, although these same genes did not reach significance in blood. Metallothioneins are known to be involved in neuroprotection against, among other things, the deleterious effects of long-term increases in cortisol levels [44]. The decreased expression of SAT1 and MT3, as well as other members of the polyamine and metallothionein families, were previously reported to be differentially expressed in MDD-S by our group [15, 24].

**Table 3:** Significantly differentially expressed genes between MDD-S and MDD-NS in the DLPFC (*p* ≤ 0.05), corresponding *q*-value after FDR correction (*q* ≤ 0.1 are in bold), and fold change in MDD-S relative to MDD-NS.

Gene	Mean (Control)	Mean (MDD-NS)	Mean (MDD-S)	*p* (MDD-S vs. MDD-NS)	*q* value (MDD-S vs. MDD-NS)	FC (MDD-S vs. MDD-NS)	FC direction
SAT1_all	11.418	11.521	11.156	0.0001	**0.005**	−1.316	DOWN
MT3	14.289	14.453	14.185	0.0002	**0.008**	−1.220	DOWN
TERC	10.105	10.241	9.898	0.001	**0.004**	−1.202	DOWN
HSD11B2	4.621	5.286	4.598	0.002	**0.004**	−1.450	DOWN
FKBP5	9.071	10.055	9.085	0.002	**0.003**	−1.592	DOWN
FGF2	10.361	10.519	10.085	0.002	**0.027**	−1.384	DOWN
MAOA	9.560	9.644	9.379	0.003	**0.013**	−1.205	DOWN
PAOX	6.324	6.440	6.219	0.003	**0.031**	−1.193	DOWN
MT2A	10.793	11.094	10.515	0.003	**0.010**	−1.432	DOWN
SAT1-002	5.685	6.021	5.673	0.004	**0.031**	−1.279	DOWN
AGMAT	6.415	6.128	6.430	0.004	**0.031**	1.244	UP
MT1A	6.737	7.591	6.228	0.004	**0.017**	−2.404	DOWN
ARNTL	10.396	10.430	10.252	0.008	**0.019**	−1.131	DOWN
SOX9	10.498	10.610	10.083	0.009	**0.027**	−1.425	DOWN
NFE2L2	10.170	10.232	9.991	0.009	**0.049**	−1.203	DOWN
ODC1	9.388	9.553	9.436	0.011	0.150	−1.107	DOWN
MT1X	9.602	10.414	9.627	0.012	**0.042**	−1.693	DOWN
PPARA	8.204	8.287	8.065	0.013	**0.075**	−1.187	DOWN
CD19	4.369	4.497	4.084	0.015	**0.019**	−1.311	DOWN
TERF1	9.958	9.998	9.908	0.015	0.150	−1.086	DOWN
RBFOX3	10.787	10.668	10.810	0.018	**0.013**	1.073	UP
CRH	8.472	7.732	8.314	0.019	**0.031**	1.352	UP
PER3	10.250	10.119	10.333	0.022	**0.031**	1.132	UP
HTR1A	6.866	7.003	6.768	0.023	0.120	−1.201	DOWN
SAT1-001	11.524	11.594	11.366	0.024	0.150	−1.189	DOWN
GRIN2C	9.267	9.268	9.072	0.030	0.180	−1.167	DOWN
OAZ2	11.797	11.874	11.800	0.043	0.226	−1.078	DOWN
SRM	10.288	10.346	10.290	0.045	0.223	−1.063	DOWN

*DLPFC* dorsolateral prefrontal cortex, *MDD-S* major depressive disorder—suicide, *MDD-NS* major depressive disorder—non-suicide, *FC* fold-change, *FDR* false discovery rate.

Comparisons were also carried out between suicide (MDD-S) and controls (Supplementary Table 9). Five genes were significantly differentially expressed in MDD-S (*p* ≤ 0.05), with the most significant, and only gene passing FDR, being SAT1. This finding provides additional evidence for the involvement of the polyamine stress response in suicide. Gene expression differences were also observed between MDD-NS and controls (Supplementary Table 10), and both MDD groups (MDD-S + MDD-NS) versus controls (Supplementary Table 11). Top genes in both these comparisons included the upregulation of stress-related genes (FKBP5, ODC1, and a splice variant of SAT1).

### Blood and brain concordant changes

Most expression changes associated with suicide were tissue specific, meaning they were observed either in blood or brain only. However, four genes showed significant changes in both brain and blood (at *p*-value ≤ 0.05, Supplementary Fig. 1). Two of these genes were concordant with regards to directionality (PER3 and SOX9). The circadian rhythm gene PER3 was significantly increased in MDD-S compared to MDD-NS (Fig. 1 and Table 3). PER3 is an important timekeeping gene involved in the regulation of circadian rhythms and is associated with delayed sleep phase disturbances in humans [45]. The other gene concordant between blood and brain was SOX9. This gene was significantly reduced in both blood and brain of MDD-S compared to MDD-NS (Figs. 1 and 2). SOX9, a marker of B cells in blood and astrocytes in brain, has previously been shown to be decreased in the frontal cortex in MDD, by our group [46], and in depressed suicides [47]. The two remaining genes, CD19 and TERF1 (Telomere Regulation factor 1), were increased in suicides in blood, while being decreased in the brain. CD19 was differentially increased in MDD-S suggesting B cell mediated inflammation in suicides. TERF1 codes for a protein that binds telomere ends and is part of the shelterin nucleoprotein complex. We [48] and others [49] have previously reported aberrant telomere length in brain and blood associated with MDD and suicide.

Cross-validation of our results with previous studies aiming to identify blood biomarkers to predict suicide risk revealed some overlapping genes. We found increased expression of SAT1 in the blood of MDD-S versus MDD-NS, although this association did not reach significance. However, when comparing MDD-S to controls we observed a significant increase of SAT1 expression associated with suicide (Supplementary Table 6). Through liquid biopsies and post-mortem validation, Niculescu et al. [35], also showed increased SAT1 levels to be associated with suicide in blood.

In our current study, and in a previous one using a cohort of mood disorder patients (MDDs and bipolar disorder) [30], we observed significantly decreased levels of the metallothionein family of genes to be associated with suicide. Similarly, Niculescu and colleagues found Metallothionein 1E (MT1E) among their universal Bonferroni validated biomarkers for suicide [35]. Lastly, the SKA2 (Spindle and Kinetochore Associated Complex Subunit 2) gene was found to be down-regulated in suicide, in both our blood and brain samples, as well as in Niculescu’s blood samples [35].

## Discussion

A targeted gene expression approach was developed to assess relevant suicide specific biomarkers in postmortem blood and brain using a platform designed to overcome any bias introduced due to sample degradation (NanoString). We used frozen blood, not preserved in specialized RNA stabilizing buffers, because of its availability and common use by Coroners and Medical Examiners for toxicology. Our results suggest non-preserved blood can be used to discover suicide specific biomarkers that can also be assessed in brain tissue from the same subjects when available. Little is known about how gene expression levels in brain directly correlate with expression changes in blood, because brain and blood samples are not usually obtained from the same subject. To our knowledge, this is the first study to determine suicide specific gene expression changes both in blood and brain tissue from the same MDD subjects to develop a molecular signature in blood to predict individuals at highest risk for suicide. We clearly show that among MDD patients, suicide victims have a gene expression signature distinct from non-suicides that includes key genes involved in stress response and polyamine metabolism, immune response, and telomere maintenance. Genes identified as dysregulated in suicide could be potential targets for future pharmacological interventions to prevent suicide in MDD patients but could also be used to develop a molecular test to identify patients at high risk for suicide.

Previous studies have predominantly focused on subjects with suicidal behaviors, but not on suicide victims, to identify state (epigenetic, expression) or trait markers for suicide by performing genome wide association studies (GWAS) in samples of patients with suicidal ideation or suicide attempts [50–59]. GWAS studies in particular have identified single nucleotide polymorphisms (SNPs) associated with suicide attempts and suicidal behaviors [50–53, 56, 58, 60]. These SNP associations while informative for suicidal behaviors are not necessarily helpful for the identification of subjects about to commit suicide. Subjects with suicidal behaviors, including attempters might have a different clinical presentation and represent a distinct phenotype that does not always predict future suicide. This makes the study of actual suicide patients particularly important for the identification, understanding and prediction of at-risk individuals.

Recent studies have shown suicide specific molecular signatures can be found in blood and brain tissue. Four recent genome wide gene association studies (GWAS) of suicide were conducted using blood [58, 61–63]. These studies, while showing a high SNP-based heritability, in large admixed populations across psychiatric diagnoses, revealed no overlap in the identification of genes associated with completed suicide. However, the association of the HTR2A (5-Hydroxytryptamine Receptor 2A) gene with death by suicide identified by Coon and colleagues [63] is corroborated by our observation of decreased HTR2A expression in MDD-S blood when compared to controls (Supplementary Table 6). Several recent investigations of gene expression were also conducted in brain tissue from suicide victims. Nagy et al. [64] performed single nuclei gene expression analysis in DLPFC from MDD suicide completers compared to controls, and found 96 differentially expressed genes and major cellular dysregulation in deep layer excitatory neurons and immature oligodendrocyte precursor cells (OPCs). Mahajan et al. [65] investigated expression changes in the hippocampus of MDDs and found significant dysregulation of immune-related genes (inflammation, cytokines) in MDDs although they did not directly compare MDD-S to MDD-NS. Finally, Jabbi et al. [66] investigated gene expression changes in suicide patients with mixed diagnoses (MDD, bipolar disorder [BD]) in the insula. Their results also showed changes in suicides vs. controls mainly related to immune function and cytokines. Overall, these recent GWAS and gene expression studies did not directly compare patients, who died by suicide to patients who died of other causes.

Comparisons have previously been made between gene expression in the brain and peripheral blood, with both tissues sharing significant transcriptomic expression [67, 68]. However, the correlation is approximately 0.5, indicating that not all genes are expressed in the same direction between both tissues. For example, SAT1, which has been consistently reported to be significantly down-regulated in the suicide brain [16, 20, 24], was found to be increased in living bipolar subjects with suicidal ideation [27], and in two groups of post-mortem blood from all-male suicide completers with mixed diagnoses (schizophrenia, bipolar, and MDD) [26, 35]. Additional evidence also shows isoform-specific differential expression of SAT1 and risk allele association with suicide [16, 20, 24]. In our study we minimized diagnostic variability by using only MDD subjects and observed a significant decrease in SAT1 expression in the brain, although SAT1 was increased in MDD-S (vs. MDD-NS), in blood, it did not reach significance. Altogether, this evidence suggests that SAT1 has the potential to be both an indicator of suicidal ideation (SI) across certain psychiatric mood disorders, and be a predictor of suicide completion that is amenable to pharmacological intervention.

Our previous work shows significantly decreased MT1E expression in both the Nucleus Accumbens and anterior cingulate cortex of mood disorder patients who died by suicide compared to non-suicides [30], while Niculescu and colleagues have observed increased MT1E expression to be among their validated universal biomarkers associated with suicidality in blood [35]. This suggests metallothionein genes could be involved in suicide biology; however, further investigation is needed to determine if select MT genes can identify patients at risk for suicide and/or predict suicide completion. Lastly, another gene characterized by Niculescu and colleagues [35] as a universal biomarker for suicide is SKA2, which was downregulated. In our cohort, this gene had decreased expression in MDD-S compared to MDD-NS but, did not reach statistical significance. Overall, there is some validation of the blood-based findings between our study and that by Niculescu and colleagues [35] (SAT1, SKA2, and metallothionein family genes) even with the methodological differences between the studies.

As described in the “Results” section, four genes showed highly significant differential expression in blood and brain. Two of these genes had similar directionality (PER3 and SOX9) and two had opposing directions (CD19 and TERF1) between blood and brain (Supplementary Fig. 1). The variability in direction between the two tissues is understandable because blood and brain may not be exposed to the same metabolic environment or stress related factors, which could influence the expression levels for some genes. SOX9 is an astrocytic marker in the brain and B cell marker in blood that has previously been shown to be decreased in MDD-S compared to controls in the prefrontal cortex [47]. Our results show that SOX9 expression is significantly reduced both in blood and brain in MDD-S compared to MDD-NS suggesting similar immune/astrocytic dysregulations in suicide that could be further investigated.

We observed a significant effect of time of death in the DLPFC. Therefore, we corrected for time of death based on hours since sunrise (adjusted for daylight savings time). The adjustment of the time of death to hours from sunrise was done by counting the number of hours between sunrise and time of death taking into account daylight saving time. Despite the correction for multiple testing, the increase in the circadian gene, PER3, still remained. Dysregulated circadian rhythms in MDD involve significant disruptions in 24 h rhythms of mood, temperature, hormone secretion and sleep [45]. The circadian system is regulated by a set of core clock genes. The period genes are central to circadian timing and provide negative feedback to shut off their own transcription through a set of complex feedback loops. Mutations in the period gene, PER3, are implicated in sleep disorders associated with shifts in circadian rhythms and are thought to increase susceptibility to MDD [69]. In postmortem brain, we studied ~12,000 genes and observed a dramatic disruption in sinusoidal rhythms of core clock genes across six brain regions of MDDs compared to non-psychiatric controls [70], with PER3 as one of the top dysregulated genes. PER3 contains a glucocorticoid recognition element (GRE) on its promoter [71] and relevant to MDD, SNPs in the PER3 gene can alter multiple systems including response to antidepressants [72]. Additional evidence for the involvement of period genes with suicide has been found by Levy et al. [28], where they identified increased blood expression of PER1 to be related to suicidality in females.

Inflammation is a frequent feature of the pathophysiology of depression with markers of inflammation observed in blood [73–76] and brain [77]. However, it is not clear if inflammation is a mediatory mechanism of depression or suicide because other factors such as obesity and smoking also play a role [73]. Our results show that peripheral postmortem blood of MDD-S has significantly higher levels of two inflammatory markers compared to MDD-NS. The expression of CD19 and CD6 genes, markers for B and T cells respectively, are upregulated (Fig. 1 and Table 2). Similarly, expression of the glucocorticoid receptor is also increased in MDD-S compared to MDD-NS (Table 2), underlining stress/inflammation upregulation in suicides. Our results are indicative of a significant relationship between suicide and an overactive immune system in MDD patients leading to the hypothesis that inflammation is playing a role in the suicidal tendencies of depressed individuals. The DLPFC also showed a similar pattern of immune dysregulation and stress response alterations in suicides. Several polyamine (PAOX, ODC1, SAT1, and AGMAT) and metallothionein (MT2A and MT1A) genes were altered in MDD-S (Table 3) confirming previous reports [16, 17, 24–27, 78, 79]. Furthermore, key inflammation and stress response genes were similarly differentially expressed in the DLPFC of suicides including cortisol related genes (CRH, HSD11B2, and FKBP5) as well as CD19 and the astrocytic marker, SOX9 [80].

Another significant finding is the involvement of several mitochondrial genes in suicide. MAOA, a gene that encodes mitochondrial enzymes, was significantly lower in the DLPFC of MDD-S compared to MDD-NS. In blood, three genes were differentially expressed between MDD-S and MDD-NS. Two nuclear genes coding for mitochondria located proteins MTPAP (a mitochondrial Poly(A) polymerase) and the mitochondrial polyamine transporter SLC25A26 were increased in blood in suicides, while expression of ND6 (a mitochondria encoded subunit of the respiratory chain Complex I) was decreased in suicides. MTPAP and SLC25A26 were also significantly increased in MDD-S compared to controls suggesting that mitochondrial alterations could be used as potential signatures to differentiate MDD-S from MDD-NS patients and also from controls.

Our study results have some limitations. First, our sample has a higher proportion of males than females. To minimize the effects of gender bias, we balanced the genders between diagnostic groups. However, it is known that a greater number of women suffer from depression and future studies could benefit from increasing the number of females. Additionally, while this a small study, it clearly shows that this approach can be useful in identifying suicide (state) biomarkers that can be used in a clinical setting to identify MDD patients in a highly suicidal state for additional interventions. In the future, larger studies of SNP (trait) markers could be combined with investigations of gene expression suicide phenotype signatures (state) to provide a more comprehensive overview of the contribution of these factors to an increased risk for suicide.

We have performed a targeted gene expression investigation of 117 genes. Although there was substantial a priori reasons for gene selection, it is quite possible that additional suicide biomarkers may have been missed because a higher throughput technology, such as RNA-Seq, was not employed. This would be a next step in future studies to confirm our current findings and to identify additional suicide-related gene expression differences. Lastly, we have only analyzed one brain region. Although the DLPFC is implicated in MDD and suicide there may be other regions, which could provide additional gene expression information (e.g., the anterior cingulate or hippocampus), to help refine our blood suicide signature. In addition, validation of the identified blood-based suicide marker could benefit from testing in a clinical sample of MDDs with severe suicidal behaviors and ideation. Multiple testing correction using methods such as FDR is useful to determine the most likely results to be differentially expressed given the number of comparisons. Because this is an exploratory study, we present and discuss both the top significant genes and the adjusted *q*-values as potential biomarkers to be tested in future clinical studies. Validation would confirm the utility of the biomarker to identify at-risk individuals who would benefit from immediate interventions to prevent suicide.

Additional studies to compare the immune cell phenotype and function in the blood of depressed suicidal and depressed non-suicidal individuals are warranted. Activation of antigen presenting cells macrophages and dendritic cells (DCs), responsible for activation of T and B lymphocytes and the phenotype and expression of activation markers particularly CD6 and B lymphocytes could be analyzed in highly suicidal MDD patients to further investigate the role of immune dysregulation. The T activation cells can also be studied and secretion of cytokines determined by multiplexing. There has also been a link proposed between increased inflammation in MDD and decreased telomere length in depression, although this has not yet been extended to encompass suicide. The relationship between inflammation and telomere length in our current sample needs to be further explored and compared between blood and brain, particularly in view of the increased levels of TERF1 in blood from patients who died by suicide (Table 2). TERF1 is of particular interest because it plays a direct role in telomere length as part of a protein complex (shelterin) responsible for protecting telomeres [81].

Overall, brain and blood differentially expressed genes were observed between MDD-S and MDD-NS. We included comparisons, in brain and blood, with the Control group and both MDD groups (Control vs. MDD-NS and Control vs. MDD-S) as well as a comparison between Controls and both MDD groups regardless of the suicide status (Control vs. MDD (S + NS) to assess gene expression changes associated with depression (Supplementary Tables 6–11).

In conclusion, our results suggest that blood expression profiles could help to identify individuals at the highest risk of death by suicide and be highly informative to understand molecular changes in suicide. Our study design, which includes age and gender matched comparison groups, allowed for the identification of suicide specific expression changes either unique to or in common between DLPFC and blood. Specifically, stress response changes, including polyamine metabolism, circadian rhythm, immune dysregulation, and telomere maintenance are shared among suicides in blood suggesting future studies in large clinical populations investigating these systems might help in the identification of acutely suicidal MDD patients.

## Supplementary information

Supplementary Information
