# Supplementary material for: Identification of potential blood biomarkers associated with suicide in major depressive disorder
Source: Transl Psychiatry. 2022 Apr 14;12:159. doi: 10.1038/s41398-022-01918-w (PMC9010430; doi:10.1038/s41398-022-01918-w)
Supplement: Supplementary file 1 — Supplementary Information [file 41398_2022_1918_MOESM1_ESM.pdf]

Supplementary Information for:

Identification of Potential Blood Biomarkers Associated with Suicide in Major Depressive Disorder

Firoza Mamdani Ph.D[1], Matthieu D. Weber B.Sc.[1], Blynn Bunney Ph.D[1], Kathleen Burke[1], Preston Cartagena Ph.D[1], David Walsh Ph.D[1], Francis S. Lee M.D., Ph.D[2], Jack Barchas M.D.[2], Alan F. Schatzberg M.D.[3], Richard Myers Ph.D[4], Stanley J. Watson M.D., Ph.D[5], Huda Akil Ph.D[5], Marquis P. Vawter Ph.D[1], William E. Bunney M.D.[1], Adolfo Sequeira Ph.D[1*]

1. Psychiatry and Human Behavior, University of California, Irvine, Irvine, CA, USA
2. Department of Psychiatry, Weill Cornell Medical College, New York, NY, USA
3. Department of Psychiatry and Behavioral Sciences, Stanford University, Palo Alto, CA, USA
4. Hudson Alpha Institute for Biotechnology, Huntsville, AL, USA
5. Molecular & Behavioral Neuroscience Institute, University of Michigan, Ann Arbor, MI, USA

Supplementary Table 1: Demographic details of the study cohort

Tissue	Gender	Age	Status	Race	pH	Blood	DLPFC	Manner of Death
Blood and Brain	F	65	Control	Caucasian	6.49	x	x	Pulmonary Embolism
	M	49	Control	Caucasian	6.5	x	x	Coronary Artery Disease
	M	53	Control	Hispanic	6.55	x	x	Severe Hypertrophic Cardiomyopathy
	M	62	Control	Pacific Islander	6.36	x	x	Coronary Atherosclerosis
	M	27	Control	Caucasian	6.55	x	x	Accident
	F	32	Control	Caucasian	6.01	x	x	Undetermined - Natural Causes
	M	21	Control	African American	6.2	x	x	Pulmonary Embolism
	M	45	Control	Caucasian	6.49	x	x	Dilated Cardiomyopathy
	M	65	Control	Caucasian	6.41	x	x	Coronary Atherosclerosis
	M	40	Control	Caucasian	6.45	x	x	Accident
	M	36	Control	Caucasian	6.19	x	x	Coronary Atherosclerosis
	M	33	Control	Caucasian	6.35	x	x	Cardiomyopathy
	M	38	Control	Caucasian	6.53	x	x	Cardiomyopathy
	F	38	Control	Asian	6.71	x	x	Aortic Dissection
	M	49	MDD-NS	Caucasian	6.66	x	x	Cardiovascular Disease
	F	52	MDD-NS	Caucasian	6.65	x	x	Acute Pancreatitis
	M	47	MDD-NS	Caucasian	6.98	x	x	Cardiomyopathy
	M	44	MDD-NS	Caucasian	6.87	x	x	Coronary Atherosclerosis
	M	38	MDD-NS	Caucasian	6.72	x	x	Acute Pancreatitis
	F	81	MDD-NS	Caucasian	6.31	x	x	Long-term medical condition
	F	59	MDD-NS	Hispanic	6.4	x	x	Long-term medical condition
	M	58	MDD-NS	Caucasian	6.79	x	x	Cardiovascular Disease
	M	44	MDD-NS	Caucasian	6.66	x	x	Accident
	M	56	MDD-NS	Caucasian	6.58	x	x	Pneumonia
	M	50	MDD-NS	Caucasian	6.41	x	x	Aortic Dissection
	F	48	MDD-NS	Caucasian	6.02	x	x	Accident
	F	41	MDD-NS	Caucasian	6.42	x	x	Long-term medical condition
	F	51	MDD-NS	Caucasian	6.37	x	x	Long-term medical condition
	F	57	MDD-NS	Caucasian	6.18	x	x	Heart Attack
	M	60	MDD-NS	Caucasian	6.4	x	x	Aneurysm
	M	62	MDD-NS	Caucasian	6.34	x	x	Strangulated Umbilical Hernia
	M	60	MDD-S	Asian	6.8	x	x	Hanging
	M	50	MDD-S	Caucasian	6.97	x	x	Hanging
	M	60	MDD-S	Caucasian	6.69	x	x	Hanging
	F	19	MDD-S	Hispanic	6.66	x	x	Hanging
	F	43	MDD-S	Caucasian	6.52	x	x	Stab Wound
	M	33	MDD-S	Asian	6.62	x	x	Carbon Monoxide
	M	55	MDD-S	Caucasian	6.52	x	x	Gunshot Wound
	M	25	MDD-S	Caucasian	6.23	x	x	Hanging
	F	39	MDD-S	Caucasian	6.81	x	x	Hanging
	F	21	MDD-S	Hispanic	6.25	x	x	Hanging
	M	27	MDD-S	Caucasian	6.46	x	x	Hanging
	M	50	MDD-S	Caucasian	6.79	x	x	Hanging
	F	31	MDD-S	Caucasian	6.62	x	x	Suicide
	F	38	MDD-S	Caucasian	6.16	x	x	Overdose

Supplementary Table 1 Continued:

Tissue	Gender	Age	Status	Race	pH	Blood	DLPFC	Manner of Death
Brain	F	55	Control	Caucasian	6.71		x	Sudden medical condition
	M	63	Control	Caucasian	6.58		x	Sudden medical condition
	M	30	Control	Hispanic	6.71		x	Sudden medical condition
	M	54	Control	Caucasian	6.39		x	Sudden medical condition
	M	51	Control	Caucasian	6.94		x	Sudden medical condition
	M	55	Control	Caucasian	6.74		x	Sudden medical condition
	M	55	Control	Caucasian	6.18		x	Accident
	F	18	MDD-NS	Caucasian	6.31		x	Sudden medical condition
	F	64	MDD-NS	Caucasian	6.89		x	Sudden medical condition
	F	44	MDD-NS	Caucasian	6.31		x	Sudden medical condition
	M	50	MDD-NS	Caucasian	6.42		x	Sudden medical condition
	F	57	MDD-NS	Caucasian	6.75		x	Undetermined/Accidental overdose
	F	49	MDD-NS	Caucasian	6.6		x	Undetermined/Accidental overdose
	F	41	MDD-NS	Caucasian	6.7		x	Accident
	M	48	MDD-S	Caucasian	6.81		x	Suicide
	M	33	MDD-S	Caucasian	6.87		x	Suicide
	F	77	MDD-S	Asian	6.67		x	Suicide
	F	19	MDD-S	Caucasian	6.7		x	Suicide
	F	42	MDD-S	Caucasian	6.69		x	Suicide
	F	40	MDD-S	Caucasian	6.47		x	Suicide
Blood	F	42	Control	African American		x		Sudden medical condition
	M	48	Control	Caucasian		x		Sudden medical condition
	M	58	MDD-NS	Caucasian		x		Sudden medical condition
	M	39	MDD-S	Caucasian		x		Suicide
	M	54	MDD-S	Caucasian		x		Suicide
	F	65	MDD-S	Caucasian		x		Suicide
	M	30	MDD-S	Caucasian		x		Suicide
	M	31	MDD-S	Caucasian		x		Accident

Supplementary Table 2: Average brain quality measures (pH, RNA Integrity Number (RIN), and Post-mortem interval (PMI)) across groups

	pH		RIN		PMI	
	**Average**	**Stdev**	**Average**	**Stdev**	**Average**	**Stdev**
**Control**	6.48	0.22	7.49	2.47	22.54	8.19
**MDD-NS**	6.53	0.24	6.93	3.29	23.61	7.70
**MDD-S**	6.62	0.22	8.42	1.81	26.90	8.71

Supplementary Table 3: NanoString gene targets, accession number, probe position, and supporting references

Group	Gene Symbol	Gene Name	Accession	Position	Target Sequence	References
Telomere Length	ACD	adrenocortical dysplasia homolog	NM_001082486	219-318	TGTCTGACGGGACCCACAGTGTCCGATGCCTGGTGACGCGGGAGGCCCTGGACACCTCGGACTGGGAGGAGAAGGAGTTCGGCTTCCGCGGGA	Mamdani F, Rollins B, et al. (2015). Transl Psychiatry, **5**(9):e636; Rice C, et al. (2016). Comput Struct Biotechnol J, **14**:161-167; Otsuka I, et al. (2017). Sci Rep, **7**(1):3176.
	CTC1	CST telomere replication complex component 1	NM_025099.5	1845-1944	TGTCTGCTGCCCTCTGCCTTCTGCCCAGCCCAGGTTTTACTTGGGGTTCTGGTGGCTTCATCTCATAAAGGTTGTCTGCAACTTCGGGACCAAAGTG	
	DKC1	dyskerin pseudouridine synthase 1	NM_001363.3	2256-2355	GATGGGCCTGGTAGTTTTCCATCTTGTTCTGGCCTAGAGGTCAGTCCTTTGCACTTCCTCAAAGCTTGTGTACAGTGCTCACCTAAATCCATCTGACT	
	GAR1	GAR1 ribonucleoprotein	NM_032993.2	642-741	AGGTGGAGGTGGGGGCTTCAGAGGAGGAAGAGGTGGTGGTTTCAGAGGGAGAGGACATTAAGTGAAACAGTTGACAGACATCACCAGTTGAC	
	NHP2	NHP2 ribonucleoprotein	NM_017838.3	316-415	AGCAGATTCGGCGCGGGGTGAAAGAGGTTCAGAAATTTGTCAACAAAGGAGAAAAAGGGATCATGGTTTTGGCAGGAGACACACTGCCCATTG	
	NOP10	NOP10 ribonucleoprotein	NM_018648.3	327-426	GGAGACTCCGTAACCAAACTCTTCGGACTGTGAGCCCTGATGCCTTTTTGCCAGCCATACTCTTTGGCATCCAGTCTCTCGTGGCGATTGATTATGC	
	OBFC1	oligonucleotide/oligosaccharide binding fold containing 1	XM_006717976.	2821-2920	GGCAGTAAGCATTCGTACATAAACAACTGTGCAGTCCCACAGGGAGACCAAGAGAATGGTGCAGTAGCCTCTGAGGAGTTTTATTTGGTTCATGC	
	PIF1	PIF1 5'-to-3' DNA helicase	NM_025049.3	733-832	CTGTGCTGAGGGCCGTCCTGAAAGGCCAGAGCATCTTCTTCACTGGGAGTGCAGGAACAGGGAAGTCATATCTGCTAAAGCGAATCCTGGGCTC	
	POT1	protection of telomeres 1	NM_015450.2	1076-1175	ATGCAGTATTTTGACCTGACTTGTCAGCTCTTGGGCAAAGCAGAAGTGGACGGAGCATCATTTCTTCTAAAGGTATGGGATGGCACCAGGACACC	
	RTEL	regulator of telomere elongation helicase 1	NM_016434.3	4746-4845	GAGAAGCCCTGAGCTACCTTGGGGTCTGGGGTGGGTTTCTGGGAAAGTGCTTCCCCAGAACTTCCCTGGCTCCTGGCCTGTGAGTGGTGCCACAC	
	TERC	telomerase RNA component	NR_001566.1	88-187	CCCGCGCGCTGTTTTTCTCGCTGACTTTCAGCGGGCGGAAAAGCCTCGGCCTGCCGCCTTCCACCGTTCATTCTAGAGCAAACAAAAAATGTCAGC	
	TERF1	telomeric repeat binding factor 1	NM_003218.3	1038-1137	CTGAAAGCAGAATACCTGTTTCAAAGAGTCAGCCGGTAACTCCTGAAAAACATCGAGCTAGAAAAAGACAGGCATGGCTTTGGGAAGAAGACAA	
	TERF2	telomeric repeat binding factor 2	NM_005652.2	2466-2565	TGACATCAACCCTAGACCTGTCTGCCTTGGCATTTGCTGTCAACATCTGCTGGGCTATGTAGGCAGGTTAATCCTCCACTTCTCATGTGGTTGAACCA	
	TERF2IP	TERF2 interacting protein	NM_018975.2	1381-1480	TTGCATTGGAACTGGCACTTATTTTCTGACCATCGCTGCTGTTGCTCTGTGAGTCCTAGATTTTTGTAGCCAAGCAGAGTTGTAGAGGGGGATAAAA	
	TERT	telomerase reverse transcriptase	NM_198255.2	2138-2237	GTGCTGCGTGTGCGGGCCCAGGACCCGCCGCCTGAGCTGTACTTTGTCAAGGACAGGCTCACGGAGGTCATCGCCAGCATCATCAAACCCCAGAA	
	TINF2	TERF1 interacting nuclear factor 2	NM_001099274	1685-1784	CTAGACCTTTGTGATAGAACTAAAATGCTCTCTGTACTCTAGTCTCCTGCCTCCTCAGCTCTGCAAGTAGTTTAGTAGGAATGAAGTGGAAGTCCAG	
Transcription & Cell type	ADCY1	adenylate cyclase 1	NM_021116.2	12106-1220	GAATGGAGAGTGATGTGGGCAAGGGTGTCATTTTCTCGCACAATACAACTCACTGAGGATGCTTCTGTAGAAGTGAGAAACACGATGAGTACAT	Nagy C, et al. (2015). Mol Psychiatry, **20**(3):320-328; Tomita H, et al. (2013). Front Genet, **4**:297; Pandey GN, et al. (2016). Int J Neuropsychopharmacol, **19**(8):pyw015.
	ANK3	ankyrin 3	NM_001149.2	1561-1660	CAGGCCCAGCCTGTTCCAGATGAAATTGTGAAAAAGATCCTTGGAAACAAAGCAACTTTTAGCCCAATTGTCACTGTGGAACCAAGAAGACGGAA	
	CACNA1C	calcium voltage-gated channel subunit alpha1 C	NM_199460.2	4786-4885	TGAAACACCCTGTGGTAGCAGCTTTGCTGTCTTCTACTTCATCAGCTTCTACATGCTCTGTGCCTTCCTGATCATCAACCTCTTTGTAGCTGTCATCAT	
	CACNB2	calcium voltage-gated channel auxiliary subunit beta 2	NM_000724.3	2611-2710	TCTTTTGTAAGTGCTACATAAATTGGCCTGGTATGGCTGCAGTCCTCCGGTTGCATACTGGACTCTTCAAAAACTGTTTTGGGTAGCTGCCACTTGA	
	CREB1	cAMP responsive element binding protein 1	NM_134442.2	201-300	CCGAGAACCAGCAGAGTGGAGATGCAGCTGTAACAGAAGCTGAAAACCAACAAATGACAGTTCAAGCCCAGCCACAGATTGCCACATTAGCCCA	
	DLG4	discs large MAGUK scaffold protein 4	NM_001365.3	2461-2560	TGCCCTGAAGAATGCGGGTCAGACGGTCACGATCATCGCTCAGTATAAACCAGAAGAGTACAGCCGATTCGAGGCCAAGATCCACGACCTTCGG	
	GFAP	glial fibrillary acidic protein	NM_002055.4	590-689	AAGCAGATGAAGCCACCCTGGCCCGTCTGGATCTGGAGAGGAAGATTGAGTCGCTGGAGGAGGAGATCCGGTTCTTGAGGAAGATCCACGAGC	
	GPHN	gephyrin	NM_020806.4	1146-1245	ACTAACCACGACCATCAAATCCGTGTCGGAGTCCTTACAGTGAGTGATAGTTGCTTCAGGAATCTTGCAGAAGACCGCAGTGGGATAAATCTCAA	
	GPR37	G protein-coupled receptor 37	NM_005302.2	1891-1990	GCTCCGGCAGAAAGGTGCATTATTAAGATCTCTCCTGATTTACCAGACACCATCTATGTTCTAGCCCTCACCTACGACAGTGCGAGACTGTGGTGG	
	MAP2	microtubule associated protein 2	NM_031845.2	5171-5270	TACTCTGTATGCTGGGATTCCGAGGTTCCAACACACTGTTACAAATCTGTGGGGGGTTTCTTTCTTCTGATAATTCTAGAGCCTGTTACCATAGAAAC	
	NFE2L2	nuclear factor, erythroid 2 like 2	NM_006164.3	996-1095	TCCCGGTCACATCGAGAGCCCAGTCTTCATTGCTACTAATCAGGCTCAGTCACCTGAAACTTCTGTTGCTCAGGTAGCCCCTGTTGATTTAGACGGT	
	RBFOX3	RNA binding protein, fox-1 homolog 3	NM_001082575	577-676	TTTAGACGTGGAGATCATTTTTAACGAGCGGGGCTCCAAGGGTTTTGGGTTTGTAACTTTTGAAACTAGCTCAGATGCTGACCGAGCCCGGGAGA	
	SKA2	spindle and kinetochore associated complex subunit 2	NM_182620.3	51-150	TTTGCACAGCAGCCAATCCTCCTGCGGCGTTTTGTGCCTTTGCCCAACAGGAAAATGTGTCTCATGCAGAGGGGCGTTACCCAGTCTCCAATGGGC	
	SOX10	SRY-box 10	NM_006941.3	2160-2259	GGCCGTGTCTCCCACTCAGGGGCTGAGAGTAGCTTTGAGGAGCCTCATTGGGGAGTGGGGGGTTCGAGGGACTTAGTGGAGTTCTCATCCCTTC	
	SOX9	SRY-box 9	NM_000346.2	2136-2235	CAGTGGCCAGGCCAACCTTGGCTAAATGGAGCAGCGAAATCAACGAGAAACTGGACTTTTTAAACCCTCTTCAGAGCAAGCGTGGAGGATGATG	

Supplementary Table 3 Continued:

Group	Gene Symbol	Gene Name	Accession	Position	Target Sequence	References
Polyamine System	AGMAT	agmatinase	NM_024758.4	2053-2152	TGCTTAGGGCTGCATCTTGGTTTTAGGACCACTGTGAACGTAAGTAAAAAGTATAAAGTAACACGGAGAGCCTAATGAAGAAACTTCTTGCTCAC	Sequeira A, et al. (2006). Arch Gen Psychiatry, **63**(1):35-48
	AZIN1	antizyme inhibitor 1	NM_148174.2	506-605	TTGCATATTCTAAGGACCCAGACATAGGCTTGGTGGCCCGTCTCTTGTCTTTCCTGGTTTATGACTTTCGGCTTTGTGGAATACGGCTGAGATGAA	
	AMD1	adenosylmethionine decarboxylase 1	NM_001634.4	811-910	TTTCCCAGAGAGTCGGGTAATCAGTCAGCCAGATCAAACCTTGGAAATTCTGATGAGTGAGCTTGACCCAGCAGTTATGGACCAGTTCTACATGA	
	EIF5A	eukaryotic translation initiation factor 5A	NM_001970.4	294-393	AATGCAGTGCTCAGCATTACGTAAGAATGGCTTTGTGGTGCTCAAAGGCCGGCCATGTAAGATCGTCGAGATGTCTACTTCGAAGACTGGCAAG	
	MTHFR	methylenetetrahydrofolate reductase	NM_005957.4	1041-1140	TGTCCAAGCTGGAGGTGCCACAGGAGATCAAGGACGTGATTGAGCCAATCAAAGACAACGATGCTGCCATCCGCAACTATGGCATCGAGCTGG	
	OAZ1	ornithine decarboxylase antizyme 1	NM_004152.2	314-413	GGTGGGCGAGGGAATAGTCAGAGGGATCACAATCTTTCAGCTAACTTATTCTACTCCGATGATCGGCTGAATGTAACAGAGGAACTAACGTCCA	
	OAZ2	ornithine decarboxylase antizyme 2	NM_002537.2	367-466	CCTTCTCTCTCAGCTCTAATATATAAGGACGAGAAGCTCACTGTGACCCAGGACCTCCCTGTGAATGATGGAAAACCTCACATCGTCCACTTCCAG	
	ODC1	ornithine decarboxylase 1	NM_002539.1	951-1050	AGACCTTCGTGCAGGCAATCTCTGATGCCCGCTGTGTTTTTGACATGGGGGCTGAGGTTGGTTTCAGCATGTATCTGCTTGATATTGGCGGTGGC	
	PAOX	polyamine oxidase	NM_152911.2	849-948	CAGGAGGCAGCCTTTCCCGGGGAGACCTTTCCAGTGTCGGTAGAGTGTGAGGATGGAGACCGGTTCCCGGCGCACCATGTCATCGTCACCGTG	
	PMF1	polyamine modulated factor 1	NM_007221.3	1023-1122	CAGCCCAGGGGCCTTTACCATGTTCTCTCCACATCCGTAAATAAACTTCCTTCACTACACTGTAATCTGTGAGCATGCTTCCTCTGCCCTGGCCTCTC	
	PMFBP1	polyamine modulated factor 1 binding protein 1	NM_031293.2	849-948	ACACTTCTCCTTGCATGATTCAAGAGCATCAGGAGACTCAGAAACGACTGTCTGAAGTCTGGCAAAAGGTCTCTCAACAGGATGATCTCATTCAA	
	SAT1-001	spermidine/spermine N1-acetyltransferase 1	ENST00000379270	326-425	CCCCTTTTACCACTGCCTGGTTGCAGAAGTGCCGAAAGAGCACTGGACTCCGGAAGGACACAGCATTGTTGGTTTTGCCATGTACTATTTTACCTA	
	SAT1-002	spermidine/spermine N1-acetyltransferase 1	ENST00000489394	316-415	GCCTGGTTGCAGAAGTGCCGAAAGAGCACTGGACTCCGGAAGGTTACAGTCTCTAGCTTCGCCATGTACATGGCCCTTCCGTGTACATGGATGG	
	SAT1-004	spermidine/spermine N1-acetyltransferase 1	ENST00000379251	248-347	GCTGGCTAAATATGAATACATGGAAGAACAAGTAATCTTAACTGAAAAAGGTAATTCAACAGTGGCGGGACGGGGGACAAGCGTTCGGTGCCT	
	SAT1_all	spermidine/spermine N1-acetyltransferase 1	NM_002970.2	463-562	ATCTTGAGGACTTCTTCGTGATGAGTGATTATAGAGGCTTTGGCATAGGATCAGAAATTCTGAAGAATCTAAGCCAGGTTGCAATGAGGTGTCG	
	SMOX	spermine oxidase	NM_175839.1	162-261	CTCAGCAGGGAAAGGTTCCTAGAAGGTGAGCGCGGACGGTATGCAAAGTTGTGAATCCAGTGGTGACAGTGCGGATGACCCTCTCAGTCGCG	
	SMS	spermine synthase	NM_004595.2	1025-1124	GGATGGGAAATATTTTACACAGGGGAACTGTGTCAATCTGACAGAAGCACTGTCGCTCTATGAAGAACAGCTGGGGCGCCTGTATTGTCCTGTG	
	SRM	spermidine synthase	NM_003132.2	255-354	GCAGTAAGACCTATGGCAACGTGCTGGTGTTGGACGGTGTCATCCAGTGCACGGAGAGAGACGAGTTCTCCTACCAGGAGATGATCGCCAACC	
Circadian Rhythm	ARNTL	aryl hydrocarbon receptor nuclear translocator like	NM_001030272.1	841-940	GATGTGACCGAGGGAAGATACTCTTTGTCTCAGAGTCTGTCTTCAAGATCCTCAACTACAGCCAGAATGATCTGATTGGTCAGAGTTTGTTTGACT	Li JZ, et al. (2013). Proc Natl Acad Sci U S A, **110**(24): 9950-9955; Melhuish Beaupre L, et al. (2020). World J Biol Psychiatry, 21(2):80-90.
	CLOCK	clock circadian regulator	NM_004898.2	2351-2450	AGCCGGAAGCATGGTCCAGATTCCATCTAGTATGCCACAAAACAGCACCCAGAGTGCTGCAGTAACTACATTCACTCAGGACAGGCAGATAAGA	
	CRY1	cryptochrome circadian clock 1	NM_004075.3	1376-1475	GAAGTGATAGAAAAGTGCACAACTCCTCTGTCTGATGACCATGATGAGAAATATGGAGTCCCTTCACTGGAAGAGCTAGGTTTTGATACAGATG	
	CRY2	cryptochrome circadian clock 2	NM_001127457.1	3326-3425	ATCACACTGACAGGCTTCTTCCTGAGATATCCTCAGGTTTTCTCAGCCAGAGAGCTGCCTTTAGAGTCCAACTGTTGTACGTATGTCACCTTCACTA	
	NPAS2	neuronal PAS domain protein 2	NM_002518.3	1459-1558	CTTTAACACACTCGACGTGGGTGCCTCGGGCCTTAATACCAGTCATTCGCCATCGGCGTCCTCAAGAAGTTCCCACAAATCCTCGCACACAGCCAT	
	PER1	period circadian clock 1	NM_002616.2	4366-4465	CCTGGACAAAGTTGCTGACAAGCTGCTGAAGTGGCCTCTCCATATTCCAGCTGAGCCTGAATCTGACTCTTGAGGGTTGGGGCTGCACTTATTTA	
	PER2	period circadian clock 2	NM_022817.2	986-1085	TGTGGAGCATGTGCAGTGGAGCAGATTCTTTTACTCAAGAATGCATGGAGGAGAAATCTTTCTTTTGCCGTGTCAGTGTCCGGAAAAGCCACGA	
	PER3	period circadian clock 3	NM_016831.1	1076-1175	GACCTGATTGGAACATCGATCCTAAGCTACCTGCACCCTGAAGATCGTTCTCTGATGGTTGCCATACACCAAAAAGTTTTGAAGTATGCAGGGCA	
Stress and Immune Response	BDNF	brain derived neurotrophic factor	NM_170732.4	731-830	CAGGGTGATGCTCAGTAGTCAAGTGCCTTTGGAGCCTCCTCTTCTCTTTCTGCTGGAGGAATACAAAAATTACCTAGATGCTGCAAACATGTCCAT	Yin H, et al. (2016). Depress Anxiety, **33**(6):531-540; Melhem NM, et al. (2017). Psychoneuroendocrinology; **77**:284-294; Tonelli LH, et al. (2008). Acta Psychiatr Scand, **117**(3):198-206.
	CRH	corticotropin releasing hormone	NM_000756.1	682-781	ACCTCCTCCGGGAAGTCTTGGAAATGGCCAGGGCCGAGCAGTTAGCACAGCAAGCTCACAGCAACAGGAAACTCATGGAGATTATTGGGAAAT	
	FKBP5	FK506 binding protein 5	NM_001145775.1	541-640	ACCATTTGTCTTTAGTCTTGGCAAAGGCCAAGTCATCAAGGCATGGGACATTGGGGTGGCTACCATGAAGAAAGGAGAGATATGCCATTTACTG	
	HSD11B1	hydroxysteroid 11-beta dehydrogenase 1	NM_181755.1	156-255	GCCTACTACTACTATTCTGCAAACGAGGAATTCAGACCAGAGATGCTCCAAGGAAAGAAAGTGATTGTCACAGGGGCCAGCAAAGGGATCGGA	
	HSD11B2	hydroxysteroid 11-beta dehydrogenase 2	NM_000196.3	1703-1802	CCTCAGGATTGTTTCTCGGCACCAGTGCCTCAGTGCTGCAATTGAGGGCTAAATCCCAAGTGTCTCTTGACTGGCTCAAGAATTAGGGCCCCAACT	
	HSP90AA1	heat shock protein 90 alpha family class A member 1	NM_001017963.2	1656-1755	CTTGACCAATGACTGGGAAGATCACTTGGCAGTGAAGCATTTTTCAGTTGAAGGACAGTTGGAATTCAGAGCCCTTCTATTTGTCCCACGACGTG	
	HSPA2	heat shock protein family A (Hsp70) member 2	NM_021979.3	2096-2195	AAGATCCTCGACAAGTGTCAGGAGGTGATCAACTGGCTCGACCGAAACCAGATGGCAGAGAAAGATGAGTATGAACACAAGCAGAAAGAGCT	
	NPY	neuropeptide Y	NM_000905.2	271-370	AGAGATATGGAAAACGATCCAGCCCAGAGACACTGATTTCAGACCTCTTGATGAGAGAAAGCACAGAAAATGTTCCCAGAACTCGGCTTGAAG	
	NR3C1	nuclear receptor subfamily 3 group C member 1	NM_001018077.1	1666-1765	GCTTTCTCCTCTGGCGGGAGAAGACGATTCATTCCTTTTGGAAGGAAACTCGAATGAGGACTGCAAGCCTCTCATTTTACCGGACACTAAACCCA	
	PPARA	peroxisome proliferator activated receptor alpha	NM_001001928.2	5221-5320	GGGTGTGTTTGCTATACGAACATAATGGACGTGAAGTGGGGCAGAAACCCAGAACTCAGCATTCAAGGATGCCCAGGAGAGCTGTCCCTGTTT	
	PPARD	peroxisome proliferator activated receptor delta	NM_006238.4	108-207	GTAGGCAGCCGGGACAGTGTTGTACAGTGTTTTGGGCATGCACGTGATACTCACACAGTGGCTTCTGCTCACCAACAGATGAAGACAGATGCAC	
	PPARG	peroxisome proliferator activated receptor gamma	NM_015869.3	1036-1135	GAGCAAAGAGGTGGCCATCCGCATCTTTCAGGGCTGCCAGTTTCGCTCCGTGGAGGCTGTGCAGGAGATCACAGAGTATGCCAAAAGCATTCCT	
	SGK1	serum/glucocorticoid regulated kinase 1	NM_005627.2	1791-1890	GTGTGAACCGTCGTGTGAGTGTGGTATGCCTGATCACAGATGGATTTTGTTATAAGCATCAATGTGACACTTGCAGGACACTACAACGTGGGAC	
	CD19	CD19 molecule	XM_011545981.1	714-813	CACCCCAAGGGGCCTAAGTCATTGCTGAGCCTAGAGCTGAAGGACGATCGCCCGGCCAGAGATATGTGGGTAATGGAGACGGGTCTGTTGTTG	
	CD6	CD6 molecule	NM_001254751.1	1723-1822	AACCCTGGACACTGCATTACAGACCCGCCATCCCTGGGCCCTCAGTATCACCCGAGGAGCAACAGTGAGTCGAGCACCTCTTCAGGGGAGGATT	
	CD68	CD68 molecule	NM_001251.2	1141-1240	ACCGGTCCATCTTGCTGCCTCTCATCATCGGCCTGATCCTTCTTGGCCTCCTCGCCCTGGTGCTTATTGCTTTCTGCATCATCCGGAGACGCCCATCC	
	CD8A	CD8a molecule	NM_001768.5	1321-1420	GCTCAGGGCTCTTTCCTCCACACCATTCAGGTCTTTCTTTCCGAGGCCCCTGTCTCAGGGTGAGGTGCTTGAGTCTCCAACGGCAAGGGAACAAGT	
	FCAR	Fc fragment of IgA receptor	NM_002000.2	1416-1515	TGCTGAGATTATAGGCATGAGCCACCACGCCTGGCCAGATGCATGTTCAAACCAATCAAATGGTGTTTTCTTATGCAGGACTGATCGATTTGCAC	
	FGF2	fibroblast growth factor 2	NM_002006.4	621-720	GTCCGGGAGAAGAGCGACCCTCACATCAAGCTACAACTTCAAGCAGAAGAGAGAGGAGTTGTGTCTATCAAAGGAGTGTGTGCTAACCGTTAC	
	FOXP3	forkhead box P3	NM_014009.3	1231-1330	GGGCCATCCTGGAGGCTCCAGAGAAGCAGCGGACACTCAATGAGATCTACCACTGGTTCACACGCATGTTTGCCTTCTTCAGAAACCATCCTGCC	
	IFNG	interferon gamma	NM_000619.2	971-1070	ATACTATCCAGTTACTGCCGGTTTGAAAATATGCCTGCAATCTGAGCCAGTGCTTTAATGGCATGTCAGACAGAACTTGAATGTGTCAGGTGACC	
	IL1B	interleukin 1 beta	NM_000576.2	841-940	GGGACCAAAGGCGGCCAGGATATAACTGACTTCACCATGCAATTTGTGTCTTCCTAAAGAGAGCTGTACCCAGAGAGTCCTGTGCTGAATGTGG	
	IL6	interleukin 6	NM_000600.3	365-464	GGCACTGGCAGAAAACAACCTGAACCTTCCAAAGATGGCTGAAAAAGATGGATGCTTCCAATCTGGATTCAATGAGGAGACTTGCCTGGTGAA	
	TNF	tumor necrosis factor	NM_000594.2	1011-1110	AGCAACAAGACCACCACTTCGAAACCTGGGATTCAGGAATGTGTGGCCTGCACAGTGAAGTGCTGGCAACCACTAAGAATTCAAACTGGGGCCT	

Supplementary Table 3 Continued:

Group	Gene Symbol	Gene Name	Accession	Position	Target Sequence	References
Neurotransmission	GABRA1	gamma-aminobutyric acid type A receptor alpha1 subunit	NM_000806.5	476-575	AAAGTCCAGGTCTGTCTGACTGTCTTTGGGCCTGGATCCTCCTTCTGAGCACACTGACTGGAAGAAGCTATGGACAGCCGTCATTACAAGATGAA	Sequeira A, et al. (2009). PLoS One, **4**(8):e6585; Yin H, et al. (2016). Am J Med Genet B Neuropsychiatr Genet, **171B**(3):414-426; Turecki G, et al. (2003). Am J Med Genet B Neuropsychiatr Genet, **118B**(1):36-40.
	GABRA2	gamma-aminobutyric acid type A receptor alpha2 subunit	NM_000807.2	751-850	CTTCGAATTCAGGATGATGGGACTCTGCTGTATACCATGAGGCTTACAGTTCAAGCTGAATGCCCAATGCACTTGGAGGATTTCCCAATGGATGC	
	GABRB3	gamma-aminobutyric acid type A receptor beta3 subunit	NM_000814.5	449-548	TGGCTGACCAGCTATGGGTGCCCGACACATATTTCTTAAATGACAAAAAGTCATTTGTGCATGGAGTGACAGTGAAAAACCGCATGATCCGTCTT	
	GABRG1	gamma-aminobutyric acid type A receptor gamma1 subunit	NM_173536.3	5921-6020	GCTTTGACTAACAGACAACATAACTGAGAGACATGGTGTGTTGGGTGGAAGGGATCCTGTAGTAGATACTTGCTTGCATGTAGATGGGTACAAA	
	GABRG2	gamma-aminobutyric acid type A receptor gamma2 subunit	NM_000816.3	1-100	GTAAGTGTGAGGGGCATGAGTATACACGAGTGTGCGTGTCTTTCCCTCTCCCTTATTTGTCTCCGTATGAGTCTCTCCTTGTACCCTCCCCCTGCCT	
	GRIA1	glutamate ionotropic receptor AMPA type subunit 1	NM_000827.3	2841-2940	ATCGAGTTCTGCTACAAATCCCGTAGTGAATCCAAGCGGATGAAGGGTTTTTGTTTGATCCCACAGCAATCCATCAACGAAGCCATACGGACATC	
	GRIA2	glutamate ionotropic receptor AMPA type subunit 2	NM_001083620.1	866-965	TGACCTATGATGCCGTTCAAGTGATGACTGAAGCCTTCCGCAACCTAAGGAAGCAAAGAATTGAAATCTCCCGAAGGGGGAATGCAGGAGACT	
	GRIA3	glutamate ionotropic receptor AMPA type subunit 3	NM_000828.4	427-526	TGCAGGAGCACAGCGCTTTCCGCTTTGCCGTGCAGTTATACAACACCAACCAGAACACCACCGAGAAGCCCTTCCATTTGAATTACCACGTAGAT	
	GRIA4	glutamate ionotropic receptor AMPA type subunit 4	NM_000829.3	476-575	GTTATTTTCTGGATTTTGGGGACTCGCCATGGGAGCCTTTCCGAGCAGCGTGCAAATAGGTGGTCTCTTCATCCGAAACACAGATCAGGAATACA	
	GRIN1	glutamate ionotropic receptor NMDA type subunit 1	NM_000832.5	1291-1390	TTCAAGAGAGTGCTGATGTCTTCCAAGTATGCGGATGGGGTGACTGGTCGCGTGGAGTTCAATGAGGATGGGGACCGGAAGTTCGCCAACTAC	
	GRIN2A	glutamate ionotropic receptor NMDA type subunit 2A	NM_000833.3	4498-4597	GAATGACAGCTATCTTCGGTCGTCCTTGAGGTCAACGGCATCGTACTGTTCCAGGGACAGTCGGGGCCACAATGATGTGTATATTTCGGAGCATG	
	GRIN2B	glutamate ionotropic receptor NMDA type subunit 2B	NM_000834.3	1145-1244	CAACACCCACGAGAAGAGAATCTACCAGTCCAATATGCTAAATAGGTATCTGATCAATGTCACTTTTGAGGGGAGGAATTTGTCCTTCAGTGAAG	
	GRIN2C	glutamate ionotropic receptor NMDA type subunit 2C	NM_000835.3	2104-2203	TTCATGATCCAAGAGCAATACATCGACACTGTGTCGGGCCTCAGTGACAAGAAGTTTCAGCGGCCTCAAGATCAGTACCCACCTTTCCGCTTCGG	
	GRIN2D	glutamate ionotropic receptor NMDA type subunit 2D	NM_000836.2	1894-1993	CATCTTCGAGTACCTCAGTCCTGTTGGTTACAACCGCAGCCTGGCCACGGGCAAGCGCCCTGGCGGTTCAACCTTCACCATTGGGAAATCCATCTG	
	GRM3	glutamate metabotropic receptor 3	NM_000840.2	461-560	TTCTGCCAAGAGTCCCAATTAGATGCGACGGCTTCAGCCTGGTCAAGGTGAAGGAAAGTTGCTTCCGCGCCTAGGAAGTGGGTTTGCCTGATAA	
	HTR1A	5-hydroxytryptamine receptor 1A	NM_000524.2	626-725	TGCTCATGCTGGTTCTCTATGGGCGCATATTCCGAGCTGCGCGCTTCCGCATCCGCAAGACGGTCAAAAAGGTGGAGAAGACCGGAGCGGACA	
	HTR2A	5-hydroxytryptamine receptor 2A	NM_000621.3	3460-3559	AGGCTGAGACGCAAGGATCACTTGAGCCCAGAAGCTCAAGGCTGCAGTGAGCCAAGTTCACACCACTGCCATTTCCTCCTGGGCAACAGAGTGA	
	MAOA	monoamine oxidase A	NM_000240.2	201-300	CGAGTATCGCGGGCCACATGTTCGACGTAGTCGTGATCGGAGGTGGCATTTCAGGACTATCTGCTGCCAAACTCTTGACTGAATATGGCGTTAGT	
	SLC6A4	solute carrier family 6 member 4	NM_001045.2	236-335	CCCAGAGATCAATTGGGATCCTTGGCAGATGGACATCAGTGTCATTTACTAACCAGCAGGATGGAGACGACGCCCTTGAATTCTCAGAAGCAGC	
Mitochondria	MFN2	mitofusin 2	NM_014874.3	2011-2110	CAGTGCTTCTCCCTCAACTATGACCTAAACTGTGACAAGCTGTGTGCTGACTTCCAGGAAGACATTGAGTTCCATTTCTCTCTCGGATGGACCATG	Rollins B, et al. (2009). PLoS One, **4**(3):e4913; Mamdani F, et al. (2014). Schizophr Res, **159**(2-3):370-375.
	MT-ATP6	mitochondrially encoded ATP synthase membrane subunit 6	ENST00000361899.1	472-571	GTACGCCTAACCGCTAACATTACTGCAGGCCACCTACTCATGCACCTAATTGGAAGCGCCACCCTAGCAATATCAACCATTAACCTTCCCTCTACAC	
	MT-ND6	mitochondrially encoded NADH dehydrogenase 6	Mito_JCM__011.1	266-365	CTTTGGATATACTACAGCGATGGCTATTGAGGAGTATCCTGAGGCATGGGGGTCAGGGGTTGAGGTCTTGGTGAGTGTTTTAGTGGGGTTAGCG	
	MTPAP	mitochondrial poly(A) polymerase	NM_018109.3	196-295	CAGCCTTCAGGGAGCGTGGAGACAGGCTTTGAAGACAAGATTCCCAAAAGGAGATTCTCTGAGATGCAAAATGAAAGACGAGAACAGGCACA	
	SLC25A26	solute carrier family 25 member 26	XM_006712957.1	432-531	AGCAGAGGGCACAGGTATCTGCTTCTACAAGAACATTTCAGATTTTCTCTAACATCTTATATGAAGAGGGTATCCAAGGGTTGTATCGAGGCTATA	
Metallothionein System	MT1A	metallothionein 1A	NM_005946.2	1-100	ACCAAGCCTTCCACGTGCGCCTTATAGCCTCTCAACTTCTTGCTTGGGATCTCCAACCTCACCGCGGCTCGAAATGGACCCCAACTGCTCCTGCGCC	Sequeira A, et al. (2012). PLoS One, **7**(4):e35367.
	MT1B	metallothionein 1B	NM_005947.2	1-100	CCTGCCCTGACTTCTCATATCTTGCCTAGGAACTCCAGGCTTGTCTTGGCTCCAAATGGATCCCAACTGCTCCTGCACCACAGGTGGCTCCTGTGCC	
	MT1E	metallothionein 1E	NM_175617.3	33-132	GGTGCAGGCGCGGAGCTGGGCCTCTGCGCCCGGCCCGACCTCCGTCTATAAATAGAGCAGCCAGTTGCAGGGCTCCATTCTGCTTTCCAACTGC	
	MT1F	metallothionein 1F	NM_005949.3	15-114	CTATCAAAGCAGCGGCCGGCTGTTGGGGTCCACCACGCCTTCCACCTGCCCCACTGCTTCTTCGCTTCTCTCTTGGAAAGTCCAGTCTCTCCTCGGC	
	MT1G	metallothionein 1G	NM_005950.1	215-314	GCATCTGCAAAGGGGCATCGGAGAAGTGCAGCTGCTGCGCCTGATGTCGGGACAGCCCTGCTCCCAAGTACAAATAGAGTGACCCGTAAAATCT	
	MT1H	metallothionein 1H	NM_005951.2	215-314	CATCTGCAAAGGGGCGTCAGAGAAGTGCAGCTGCTGTGCCTGATGTCGGGACAGCCCTGCTGTCAGATGAAAACAGAATGACACGTAAAATCC	
	MT1X	metallothionein 1X	NM_005952.3	32-131	TTCCTCTTGATCGGGAACTCCTGCTTCTCCTTGCCTCGAAATGGACCCCAACTGCTCCTGCTCGCCTGTTGGCTCCTGTGCCTGTGCCGGCTCCTGCA	
	MT2A	metallothionein 2A	NM_005953.3	226-325	CAGGGCTGCATCTGCAAAGGGGCGTCGGACAAGTGCAGCTGCTGCGCCTGATGCTGGGACAGCCCCGCTCCCAGATGTAAAGAACGCGACTTC	
	MT3	metallothionein 3	NM_005954.2	166-265	CACTAGCCAAGCCGCGCGTCCAGTTGCTTGGAGAAGCCCGTTCACCGCCTCCAGCTGCTGCTCTCCTCGACATGGACCCTGAGACCTGCCCCTGC	
Housekeeping	CYC1	cytochrome c1	NM_001916.4	345-444	TATCCGTGGTCTCACCGTGGCCTCCTCTCTTCCTTGGACCACACCAGCATCCGGAGGGGTTTCCAGGTATATAAGCAGGTGTGCGCCTCCTGCCAC	
	GAPDH	glyceraldehyde-3-phosphate dehydrogenase	NM_002046.3	973-1072	CACTCCTCCACCTTTGACGCTGGGGCTGGCATTGCCCTCAACGACCACTTTGTCAAGCTCATTTCCTGGTATGACAACGAATTTGGCTACAGCAACA	
	SDHA	succinate dehydrogenase complex flavoprotein subunit A	NM_004168.1	231-330	TGGAGGGGCAGGCTTGCGAGCTGCATTTGGCCTTTCTGAGGCAGGGTTTAATACAGCATGTGTTACCAAGCTGTTTCCTACCAGGTCACACACTG	

Supplementary Table 4: Top five genes affected by adjusted time of death in the DLPFC (P≤0.05)

Gene	P (Sex)	P (Age)	P (Diagnosis)	P (Adjusted TOD)	P (MDD-S vs. MDD-NS)	FC (MDD-S vs. MDD-NS)	FC Direction
PER2	0.0003	0.057	0.205	0.003	0.158	1.193	UP
CRY1	0.013	0.034	0.504	0.016	0.880	1.014	UP
CRH	0.001	0.015	0.064	0.030	0.022	1.384	UP
SAT1-002	0.115	0.283	0.035	0.039	0.649	-1.063	DOWN
SAT1-004	0.594	0.010	0.287	0.041	0.165	-1.242	DOWN

Supplementary Table 5: Field of View (FOV) for brain and blood

	Average FOV	FOV Stdev	% FOV	Min % FOV	Max % FOV
**Brain**	533.94	10.84	96.21	85.23	98.74
**Blood**	540.06	8.27	97.31	92.43	99.64

The FOV is an imaging quality control measure from NanoString which is indicative of the number of mRNA counts successfully imaged per lane in the array. Samples having low assay efficiency due to low quality of RNA will have a low number of counts and will result in a quality flag. The FOV threshold is set at 75% and any sample below this threshold is flagged and should not be used for downstream analysis. As can be seen in the table we have included in the supplemental, our % FOV is very comparable between brain and blood, thus demonstrating the lack of effect of low RIN in the blood on mRNA measures.

Supplementary Table 6: Blood differentially expressed genes between MDD-S and Controls (P≤0.05)

Gene	Mean (Control)	Mean (MDD-NS)	Mean (MDD-S)	P (MDD-S vs. Control)	Q Value (MDD-S vs. Control)	FC (MDD-S vs. Control)	FC Direction
POT1	6.624	6.886	7.403	0.0009	**0.035**	1.841	UP
SLC25A26	6.834	6.815	7.945	0.002	**0.018**	2.297	UP
EIF5A	9.528	9.873	10.272	0.004	**0.035**	1.672	UP
HTR2A	3.361	2.843	2.209	0.005	**0.019**	-2.189	DOWN
GAR1	6.209	6.227	6.734	0.006	**0.018**	1.443	UP
NR3C1	9.315	9.465	10.435	0.008	**0.036**	2.300	UP
MT-ND6	13.714	13.262	12.791	0.011	**0.035**	-1.902	DOWN
NHP2	7.803	8.034	8.413	0.013	**0.036**	1.520	UP
SRM	7.273	7.429	7.761	0.019	**0.060**	1.440	UP
MT1H	5.345	4.760	4.071	0.021	**0.035**	-2.236	DOWN
ODC1	7.747	8.723	8.643	0.021	**0.029**	1.767	UP
GPHN	7.824	7.781	8.461	0.023	**0.060**	1.646	UP
SAT1-002	6.361	7.195	7.177	0.028	**0.053**	1.855	UP
TINF2	7.347	7.500	7.780	0.029	**0.070**	1.423	UP
MTPAP	6.824	6.628	7.377	0.029	**0.046**	1.471	UP
SAT1_all	12.901	13.633	13.815	0.037	**0.053**	1.884	UP
ACD	8.306	7.556	7.724	0.038	**0.072**	-1.471	DOWN
NFE2L2	9.360	9.712	10.001	0.045	**0.072**	1.578	UP
NOP10	8.876	9.439	9.482	0.046	**0.053**	1.439	UP
GRIN2B	4.063	3.722	3.368	0.047	**0.035**	-1.579	DOWN

Supplementary Table 7: Blood differentially expressed genes between MDD-NS and Controls (P≤0.05)

Gene	Mean(Control)	Mean(MDD-NS)	Mean(MDD-S)	P (MDD-NS vs. Control)	Q Value (MDD-NS vs. Control)	FC (MDD-NS vs. Control)	FC Direction
TERC	11.089	10.318	10.666	0.013	0.226	-1.637	DOWN
ODC1	7.747	8.723	8.643	0.013	0.226	1.996	UP
ACD	8.306	7.556	7.724	0.022	0.226	-1.619	DOWN
PMFBP1	5.426	4.690	4.781	0.042	0.384	-1.657	DOWN

Supplementary Table 8: Blood differentially expressed genes between MDDs (regardless of the suicide status) and control groups (P≤0.05)

Gene	Mean(Control)	Mean(MDD-NS)	Mean(MDD-S)	P (MDD vs. Control)	Q Value (MDD vs. Control)	FC (MDD vs. Control)	FC Direction
ODC1	7.747	8.723	8.643	0.007	**0.070**	1.878	UP
ACD	8.306	7.556	7.724	0.013	**0.103**	-1.543	DOWN
POT1	6.624	6.886	7.403	0.013	0.117	1.511	UP
EIF5A	9.528	9.873	10.272	0.013	**0.103**	1.505	UP
TERC	11.089	10.318	10.666	0.019	**0.103**	-1.466	DOWN
HTR2A	3.361	2.843	2.209	0.024	**0.103**	-1.783	DOWN
MT-ND6	13.714	13.262	12.791	0.029	**0.103**	-1.664	DOWN
PMFBP1	5.426	4.690	4.781	0.032	**0.103**	-1.552	DOWN
SRM	7.273	7.429	7.761	0.038	0.191	1.346	UP
MT1H	5.345	4.760	4.071	0.040	**0.103**	-1.929	DOWN
SAT1-002	6.361	7.195	7.177	0.040	0.124	1.706	UP
NOP10	8.876	9.439	9.482	0.042	**0.103**	1.412	UP

Supplementary Table 9: DLPFC differentially expressed genes between MDD-S and Controls (P≤0.05)

Gene	Mean(Control)	Mean(MDD-NS)	Mean(MDD-S)	P (MDD-S vs. Control)	Q Value (MDD-S vs. Control)	FC (MDD-S vs. Control)	FC Direction
SAT1_all	11.418	11.521	11.156	0.007	**0.097**	-1.210	DOWN
CRY2	10.478	10.382	10.377	0.016	0.397	-1.132	DOWN
GABRA2	12.311	12.205	12.103	0.019	0.198	-1.145	DOWN
MT2A	10.793	11.094	10.515	0.032	0.286	-1.307	DOWN
MT1A	6.737	7.591	6.228	0.052	0.417	-1.841	DOWN

Supplementary Table 10: DLPFC differentially expressed genes between MDD-NS and Controls (P≤0.05)

Gene	Mean(Control)	Mean(MDD-NS)	Mean(MDD-S)	P (MDD-NS vs. Control)	Q Value (MDD-NS vs. Control)	FC (MDD-NS vs. Control)	FC Direction
FKBP5	9.071	10.055	9.085	0.001	**0.015**	1.675	UP
ODC1	9.388	9.553	9.436	0.001	**0.101**	1.149	UP
SAT1-002	5.685	6.021	5.673	0.006	0.121	1.283	UP
SMOX	9.355	9.551	9.444	0.009	0.121	1.164	UP
RBFOX3	10.787	10.668	10.810	0.010	**0.101**	-1.087	DOWN
MT3	14.289	14.453	14.185	0.011	0.264	1.151	UP
HSD11B2	4.621	5.286	4.598	0.016	**0.015**	1.353	UP
AGMAT	6.415	6.128	6.430	0.016	0.121	-1.215	DOWN
GFAP	14.073	14.490	14.103	0.017	0.264	1.438	UP
AMD1	12.012	12.083	11.998	0.035	0.336	1.099	UP
MT1H	6.509	5.765	5.965	0.042	0.179	-1.750	DOWN
CRY2	10.478	10.382	10.377	0.045	0.336	-1.111	DOWN
PPARA	8.204	8.287	8.065	0.047	0.480	1.157	UP
CRH	8.472	7.732	8.314	0.049	**0.015**	-1.310	DOWN

Supplementary Table 11: DLPFC differentially expressed genes between MDDs (regardless of the suicide status) and control groups (P≤0.05)

Gene	Mean(Control)	Mean(MDD-NS)	Mean(MDD-S)	P (MDD vs. Control)	Q Value (MDD vs. Control)	FC (MDD vs. Control)	FC Direction
CRY2	10.478	10.382	10.377	0.013	0.548	-1.121	DOWN
SMOX	9.355	9.551	9.444	0.017	0.283	1.127	UP
ODC1	9.388	9.553	9.436	0.017	0.283	1.092	UP
FKBP5	9.071	10.055	9.085	0.037	0.283	1.327	UP

Supplementary Figure 1: Distribution of RNA Integrity Numbers (RIN) in both brain (DLPFC) and blood across the three diagnostic groups (Controls, MDD-S, and MDD-NS)

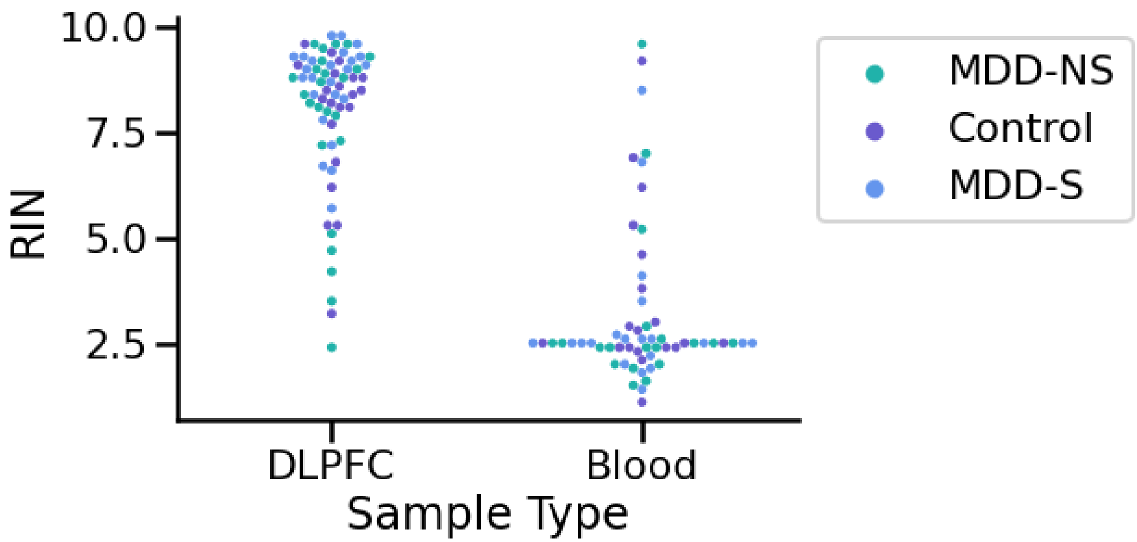

Supplementary Figure 2: Genes significantly differentially expressed, and concordant, between MDD-S and MDD-NS in blood and DLPFC. Two genes (SOX9 and PER3) are increased in suicides in both brain and blood. While two genes (CD19 and TERF1) are increased in blood, but decreased in DLPFC.

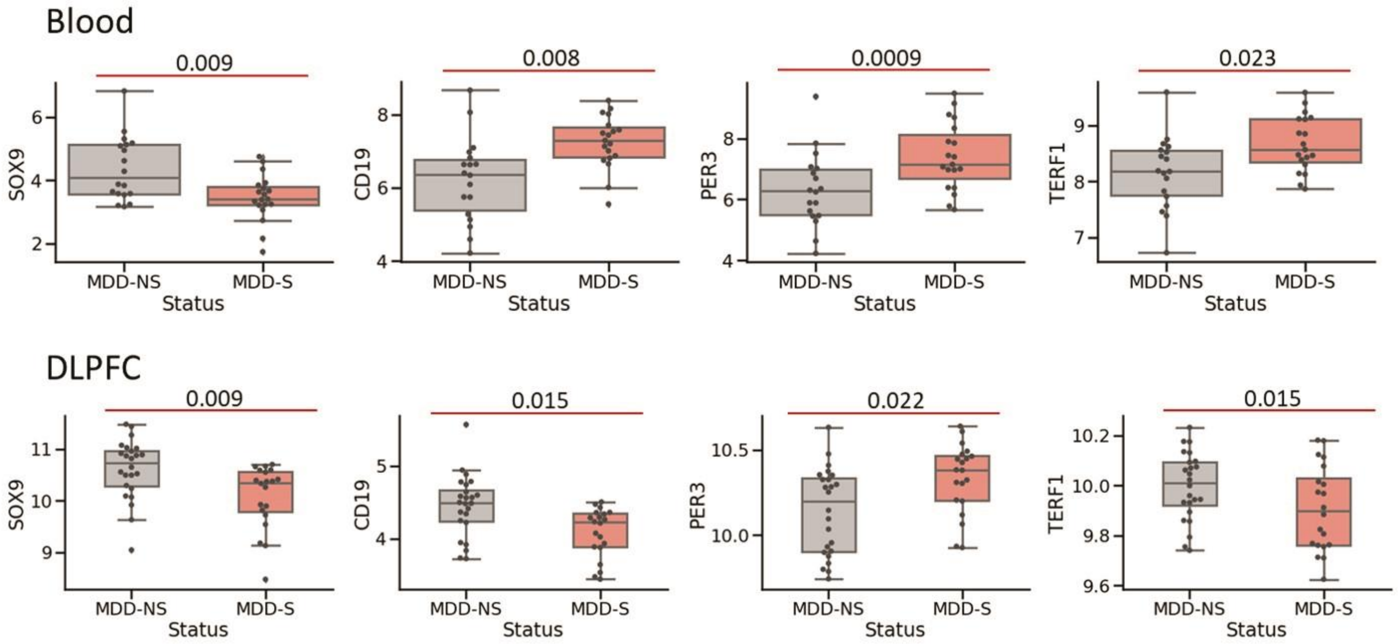

Human Brain Dissection and Freezing Protocol

Ten-Step Process for Obtaining High Quality Brain Tissue (UCI/Pritzker Brain Bank)

The brains are obtained from the Orange County Coroner's Offices with which the UCI Brain Bank has had long-standing and excellent relationships. The process involves collecting brains from schizophrenia (SZ), bipolar depressive disorder (BPD), major depressive disorder (MDD) patients, and control subjects with no psychiatric diagnoses.

1. UCI staff members identify potential brain donor candidates.

2. Prior to autopsy, all available clinical information (exclusion/inclusion criteria) is reviewed before contacting next-of-kin (NOK). The study is thoroughly explained, and consent is obtained and recorded by phone. Subsequently, the consent is obtained in writing.

3. Brain, blood, and skin are acquired during autopsy.

4. The whole brain is kept in an isotonic saline solution cooled by wet ice and taken to the UCI laboratory. Once in the lab the brain is imaged with a digital camera.

5. The brain is suspended in an alginate mold to securely hold the brain in place during slabbing. This greatly improves the consistency of slabbing so that specific brain regions are much better visualized. The brain is cortically sliced into ~12 1.5 cm slabs and each slab is digitally imaged before it is snap frozen in a -40°C dry ice cooled isopentane (2-methylbutane) bath. Brain slabs are then quickly vacuum-sealed to minimize ice crystal formation and stored in freezers in a heavily restricted area.

6. Each freezer is equipped with a sensaphone surveillance system, which automatically notifies research staff (24/7) whenever a freezer shows an increase in freezer temperature. Each freezer also has a liquid nitrogen back-up in case the freezer goes down. Finally, all freezers are individually monitored by research staff on a daily basis 365 days a year.

   The brains are stored at -80°C while evaluations are undertaken. The evaluation process includes a psychological autopsy, examination by a neuropathologist. A small piece of cerebellum from each case is analyzed for pH and RNA Integrity Number (RIN), conducted at Mark Vawter's Functional Genomics Laboratory, thus providing important data for quality control.

7. The psychological autopsy process involves information and collection of all available medical records from doctors and hospitals, and an extensive phone interview with the NOK. The NOK interview includes 140 variables. Information from this interview and the medical records are reviewed to obtain a psychiatric diagnosis by a Ph.D. and an M.D.

8. While the psychological autopsy is being completed, the digital pictures of the brain taken at the initial slabbing are sent to a neuropathologist who evaluates them for brain pathology and generates a report.

9. All cases assigned for UCI/Pritzker cohorts must fulfill the following quality control criteria:

   a) Confirmed diagnosis
   b) Completed NOK interview
   c) Negative neuropathologist report
   d) Confirmed rapid death
   e) pH >6.5
   f) RIN value > 7.0

References from Supplementary Table 2

1. Li, J. Z., B. G. Bunney, F. Meng, M. H. Hagenauer, D. M. Walsh, M. P. Vawter, S. J. Evans, P. V. Choudary, P. Cartagena, J. D. Barchas, A. F. Schatzberg, E. G. Jones, R. M. Myers, S. J. Watson, Jr., H. Akil and W. E. Bunney (2013). "Circadian patterns of gene expression in the human brain and disruption in major depressive disorder." Proc Natl Acad Sci U S A 110(24): 9950-9955.

2. Mamdani, F., B. Rollins, L. Morgan, R. M. Myers, J. D. Barchas, A. F. Schatzberg, S. J. Watson, H. Akil, S. G. Potkin, W. E. Bunney, M. P. Vawter and P. A. Sequeira (2015). "Variable telomere length across post-mortem human brain regions and specific reduction in the hippocampus of major depressive disorder." Transl Psychiatry 5: e636.

3. Mamdani, F., B. Rollins, L. Morgan, P. A. Sequeira and M. P. Vawter (2014). "The somatic common deletion in mitochondrial DNA is decreased in schizophrenia." Schizophr Res 159(2-3): 370-375.

4. elhem, N. M., S. Munroe, A. Marsland, K. Gray, D. Brent, G. Porta, A. Douaihy, M. L. Laudenslager, F. DePietro, R. Diler, H. Driscoll and P. Gopalan (2017). "Blunted HPA axis activity prior to suicide attempt and increased inflammation in attempters." Psychoneuroendocrinology 77: 284-294.

5. Melhuish Beaupre, L., G. M. Brown and J. L. Kennedy (2020). "Circadian genes in major depressive disorder." World J Biol Psychiatry 21(2): 80-90.

6. Nagy, C., M. Suderman, J. Yang, M. Szyf, N. Mechawar, C. Ernst and G. Turecki (2015). "Astrocytic abnormalities and global DNA methylation patterns in depression and suicide." Mol Psychiatry 20(3): 320-328.

7. Otsuka, I., T. Izumi, S. Boku, A. Kimura, Y. Zhang, K. Mouri, S. Okazaki, K. Shiroiwa, M. Takahashi, Y. Ueno, O. Shirakawa, I. Sora and A. Hishimoto (2017). "Aberrant telomere length and mitochondrial DNA copy number in suicide completers." Sci Rep 7(1): 3176.

8. Pandey, G. N., H. S. Rizavi, H. Zhang, R. Bhaumik and X. Ren (2016). "The Expression of the Suicide-Associated Gene SKA2 Is Decreased in the Prefrontal Cortex of Suicide Victims but Not of Nonsuicidal Patients." Int J Neuropsychopharmacol 19(8).

9. Rice, C. and E. Skordalakes (2016). "Structure and function of the telomeric CST complex." Comput Struct Biotechnol J 14: 161-167.

10. Rollins, B., M. V. Martin, L. Morgan and M. P. Vawter (2010). "Analysis of whole genome biomarker expression in blood and brain." Am J Med Genet B Neuropsychiatr Genet 153b(4): 919-936.

11. Sequeira, A., F. G. Gwadry, J. M. Ffrench-Mullen, L. Canetti, Y. Gingras, R. A. Casero, Jr., G. Rouleau, C. Benkelfat and G. Turecki (2006). "Implication of SSAT by gene expression and genetic variation in suicide and major depression." Arch Gen Psychiatry 63(1): 35-48.

12. Sequeira, A., F. Mamdani, C. Ernst, M. P. Vawter, W. E. Bunney, V. Lebel, S. Rehal, T. Klempan, A. Gratton, C. Benkelfat, G. A. Rouleau, N. Mechawar and G. Turecki (2009). "Global brain gene expression analysis links glutamatergic and GABAergic alterations to suicide and major depression." PLoS One 4(8): e6585.

13. Sequeira, A., L. Morgan, D. M. Walsh, P. M. Cartagena, P. Choudary, J. Li, A. F. Schatzberg, S. J. Watson, H. Akil, R. M. Myers, E. G. Jones, W. E. Bunney and M. P. Vawter (2012). "Gene expression changes in the prefrontal cortex, anterior cingulate cortex and nucleus accumbens of mood disorders subjects that committed suicide." PLoS One 7(4): e35367.

14. Tomita, H., M. E. Ziegler, H. B. Kim, S. J. Evans, P. V. Choudary, J. Z. Li, F. Meng, M. Dai, R. M. Myers, C. R. Neal, T. P. Speed, J. D. Barchas, A. F. Schatzberg, S. J. Watson, H. Akil, E. G. Jones, W. E. Bunney and M. P. Vawter (2013). "G protein-linked signaling pathways in bipolar and major depressive disorders." Front Genet 4: 297.

15. Tonelli, L. H., J. Stiller, D. Rujescu, I. Giegling, B. Schneider, K. Maurer, A. Schnabel, H. J. Möller, H. H. Chen and T. T. Postolache (2008). "Elevated cytokine expression in the orbitofrontal cortex of victims of suicide." Acta Psychiatr Scand 117(3): 198-206.

16. Turecki, G., A. Sequeira, Y. Gingras, M. Séguin, A. Lesage, M. Tousignant, N. Chawky, C. Vanier, O. Lipp, C. Benkelfat and G. A. Rouleau (2003). "Suicide and serotonin: study of variation at seven serotonin receptor genes in suicide completers." Am J Med Genet B Neuropsychiatr Genet 118b(1): 36-40.

17. Yin, H., H. Galfalvy, S. P. Pantazatos, Y. Y. Huang, G. B. Rosoklija, A. J. Dwork, A. Burke, V. Arango, M. A. Oquendo and J. J. Mann (2016). "GLUCOCORTICOID RECEPTOR-RELATED GENES: GENOTYPE AND BRAIN GENE EXPRESSION RELATIONSHIPS TO SUICIDE AND MAJOR DEPRESSIVE DISORDER." Depress Anxiety 33(6): 531-540.

18. Yin, H., S. P. Pantazatos, H. Galfalvy, Y. Y. Huang, G. B. Rosoklija, A. J. Dwork, A. Burke, V. Arango, M. A. Oquendo and J. J. Mann (2016). "A pilot integrative genomics study of GABA and glutamate neurotransmitter systems in suicide, suicidal behavior, and major depressive disorder." Am J Med Genet B Neuropsychiatr Genet 171b(3): 414-426.
